# Cortical Contractility Triggers a Stochastic Switch to Fast Amoeboid Cell Motility

**DOI:** 10.1016/j.cell.2015.01.008

**Published:** 2015-02-12

**Authors:** Verena Ruprecht, Stefan Wieser, Andrew Callan-Jones, Michael Smutny, Hitoshi Morita, Keisuke Sako, Vanessa Barone, Monika Ritsch-Marte, Michael Sixt, Raphaël Voituriez, Carl-Philipp Heisenberg

**Affiliations:** 1 Institute of Science and Technology Austria, 3400 Klosterneuburg, Austria; 2 Laboratoire Jean Perrin and Laboratoire de Physique Théorique de la Matière Condensée, CNRS/Université Pierre et Marie Curie, 75005 Paris, France; 3 Division of Biomedical Physics, Innsbruck Medical University, 6020 Innsbruck, Austria; 4 Laboratoire Matière et Systèmes Complexes, CNRS/Université Paris-Diderot, UMR 7057, 75204 Paris Cedex 13, France

## Abstract

3D amoeboid cell migration is central to many developmental and disease-related processes such as cancer metastasis. Here, we identify a unique prototypic amoeboid cell migration mode in early zebrafish embryos, termed stable-bleb migration. Stable-bleb cells display an invariant polarized balloon-like shape with exceptional migration speed and persistence. Progenitor cells can be reversibly transformed into stable-bleb cells irrespective of their primary fate and motile characteristics by increasing myosin II activity through biochemical or mechanical stimuli. Using a combination of theory and experiments, we show that, in stable-bleb cells, cortical contractility fluctuations trigger a stochastic switch into amoeboid motility, and a positive feedback between cortical flows and gradients in contractility maintains stable-bleb cell polarization. We further show that rearward cortical flows drive stable-bleb cell migration in various adhesive and non-adhesive environments, unraveling a highly versatile amoeboid migration phenotype.

## Highlights

• Embryonic progenitor cells transform into a prototypic amoeboid migration mode
• Contractility driven cortical network instabilities drive rapid cell polarization
• Cell polarization is maintained by a positive cortical feedback loop
• Cortical flows drive fast and persistent cell motility in confined 3D environments

## Graphical Abstract

**Figure:**
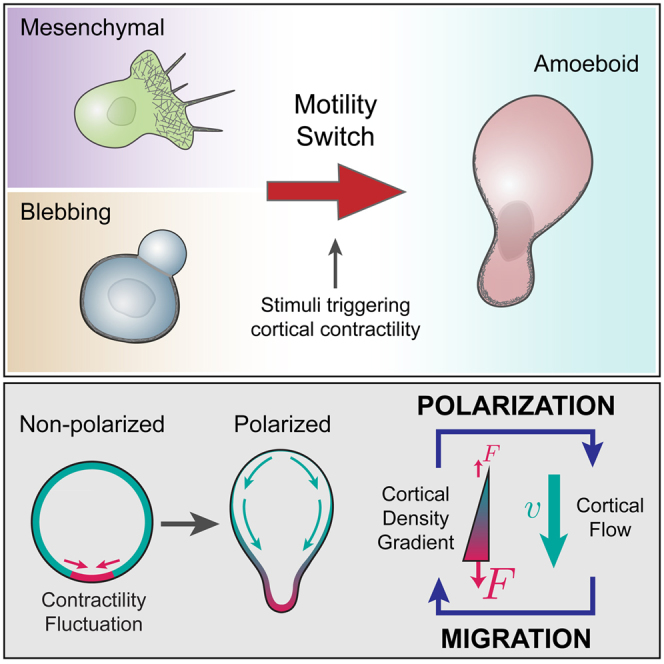

## Teaser

Cell-intrinsic fluctuation in cortical contraction forces triggers the switch in motility behavior that allows embryonic progenitor cells to acquire a fast and persistent migratory mode in confined 3D environments

## Introduction

Migrating cells show a versatile repertoire of migration modes with remarkable plasticity, allowing them to switch between different migration strategies in response to changing environmental conditions and activation of distinct molecular pathways (Friedl and Alexander, 2011). In order to migrate, cells need to establish an axis of polarity prior to movement. This polarity ultimately manifests itself in a polarized architecture of the actomyosin network, which in turn drives cell locomotion through different mechanical principles: in mesenchymal migration, the cortical actomyosin network facilitates unidirectional movement via polarized actin polymerization at the leading edge, combined with myosin-based contraction at the cell rear to disassemble adhesion sites. Amoeboid cells, in contrast, show heterogeneous shape and motility characteristics with actin-based protrusions, such as lamellipodia and pseudopodia and contraction-mediated protrusions, such as cellular blebs (Lämmermann and Sixt, 2009). Recent studies have suggested that propulsive forces in amoeboid cells are generated by cortical contractility and retrograde cortical flows (Blaser et al., 2006; Poincloux et al., 2011; Shih and Yamada, 2010), allowing movement even in the absence of specific adhesive coupling to the environment (Lämmermann and Sixt, 2009; Tozluoğlu et al., 2013).

During zebrafish gastrulation, progenitor cells become motile and undergo extensive migration to form the ectoderm, mesoderm, and endoderm germ layers. While ectodermal progenitors assemble in a pseudo-epithelial cell layer, mesodermal and endodermal (mesendodermal) progenitor cells display a highly motile mesenchymal phenotype with a mixture of lamellipodia and bleb-like protrusions (Row et al., 2011). Interfering with the ratio of those protrusion types has been shown to lower the directionality but not the speed of their migration (Diz-Muñoz et al., 2010). Besides mesendodermal progenitors, primordial germ cells (PGCs) also undergo extensive migration during gastrulation but nearly exclusively use bleb-like protrusions for their migration (Blaser et al., 2006). Although using different protrusion types, migration speed and directionality of PGCs and mesendodermal progenitors appear surprisingly similar (Blaser et al., 2006; Diz-Muñoz et al., 2010), raising questions as to the choice and benefit of certain protrusion types over others for the migration of the different progenitor cell types during gastrulation.

Here, we have studied different migration phenotypes during zebrafish gastrulation and identified a cortical contractility-mediated cell-intrinsic motility switch to fast amoeboid migration in 3D environments, which we termed stable-bleb migration.

## Results

### Identification of Basic Migration Modes in Zebrafish Germ Layer Progenitor Cells

**Figure 1:**
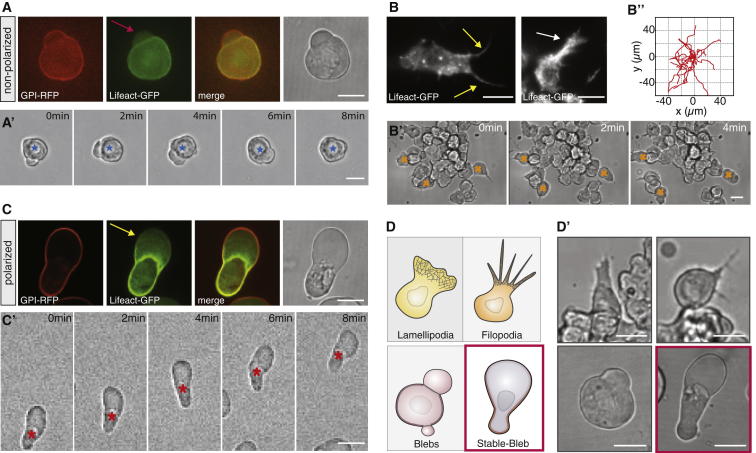
Zebrafish Germ Layer Progenitor Cells Exhibit Distinct Cell Migration Modes In Vitro (A) Bright-field (BF) and fluorescence images of blebbing progenitor cells cultured in serum-free medium in confinement with GPI-RFP (membrane, red) and Lifeact-GFP (cortex, green). Red arrow marks cellular bleb. Scale bar represents 20 μm. (A′) BF time-lapse images of non-motile progenitor cells related to culture conditions in (A). Blue asterisk indicates cell center. Scale bar represents 20 μm. (B) TIRFM images of Lifeact-GFP in mesendodermal progenitors cultured on 2D substrates. Arrows mark filopodia (yellow) and lamellipodia (white). Scale bar represents 10 μm. (B′) BF time-lapse images related to culture conditions in (B). Orange asterisks indicate exemplary migrating cells. Scale bar represents 20 μm. (B″) Representative tracks of motile mesendodermal cells over 20 min (time lag 30 s, n = 15 cells). (C) BF and fluorescence images of polarized progenitor cells with GPI-RFP (red) and Lifeact-GFP (green) cultured in 20% serum in confinement. Yellow arrow points at the bleb-like protrusion front. Scale bar represents 20 μm. (C′) BF time-lapse images of motile stable-bleb cells cultured as described in (C). Red asterisk indicates cell movement. Scale bar represents 20 μm. (D and D′) Sketch and BF images summarizing different migration phenotypes of embryonic progenitor cells in vitro. All progenitor cells were obtained from embryos at sphere stage and cultured on Fibronectin-coated substrates. Scale bar represents 20 μm. See also Movie S1 and Extended Experimental Procedures.

To study the emergence of migration competence in early germ layer progenitor cells, we aimed at developing in vitro assays to investigate the complex range of migration behaviors observed in vivo under controlled conditions with a minimal set of defined environmental parameters. Early progenitor cells placed on 2D substrates displayed a characteristic blebbing morphology that can also be observed in early blastula stage embryos in vivo (Diz-Muñoz et al., 2010). Notably, those blebbing cells failed to migrate irrespective of adhesive substrate coating with extracellular matrix (ECM) components, such as Laminin or Fibronectin (Figure 1A; Movie S1 available online). However, when progenitor cells were induced to be of mesodermal or mesendodermal origin and placed on Fibronectin-coated substrates, they formed a characteristic mixture of lamellipodia and filopodia (Figure 1B) and underwent collective migration with similar speed (<v_Cell_> = 3.8 ± 0.3 μm/min) to their movement in vivo (Figure 1B′; Movie S1).

Strikingly, when adding serum to the culture medium, we observed unexpected changes in progenitor cell architecture with cells displaying a highly polarized cell morphology characterized by a stable pear-like shape and a large spherical protrusion front (Figure 1C). These cells, which we termed stable-bleb cells, were non-motile when plated on adhesive 2D substrates, but became highly migratory in confined environments (Figure 1C′; Movie S1). Thus, in the presence of serum, progenitors transformed into a novel migration mode in vitro, clearly distinct from previously described lamellipodia-, filopodia-, and bleb-based migration types (Figure 1D).

### Changes in Cortical Architecture Precede Stable-Bleb Cell Polarization

**Figure 2:**
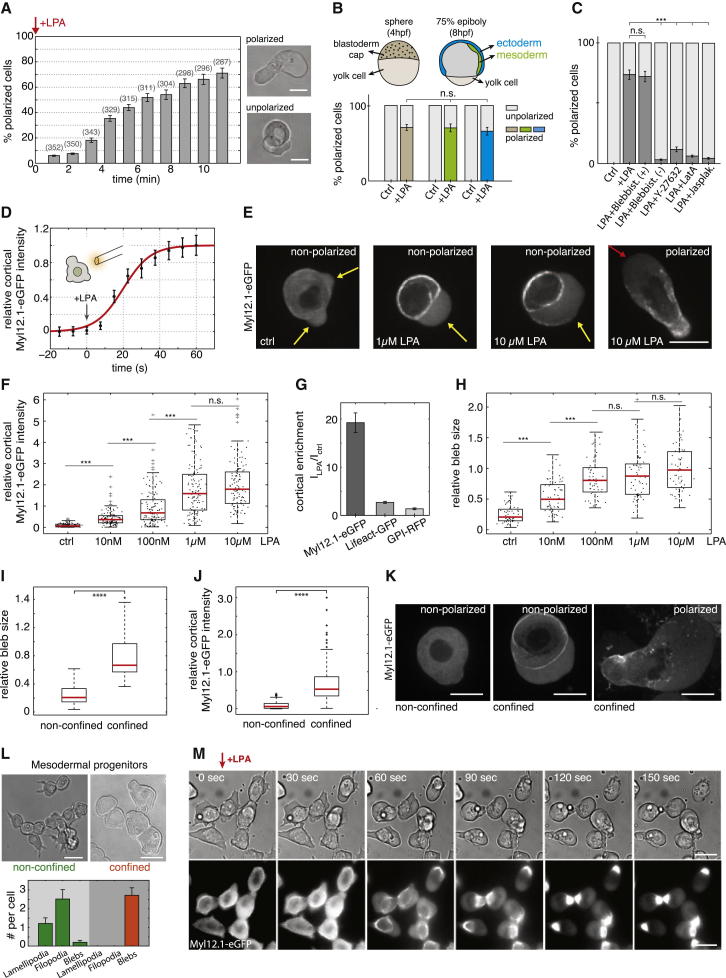
LPA Induces Cell Polarization to Stable-Bleb Cells In Vitro (A) Percentage of polarized stable-bleb cells over time upon stimulation with 100 μM LPA. (B) Percentage of polarized stable-bleb cells from blastula stage embryos (brown, n = 287) or 75% epiboly stage *Tg*(*mezzo:eGFP*) transgenic embryos with mesendodermal (green, n = 183) and ectodermal progenitors (blue, n = 152) after 10 min in the presence of 100 μM LPA (+LPA) or DMEM-F12 culture medium alone (Control [Ctrl] at blastula stage [n = 321], mesendodermal [n = 286], and ectodermal [n = 251]). (C) Percentage of polarized stable-bleb cells cultured in DMEM-F12 medium (Ctrl, n = 272), 100 μM LPA (+LPA, n = 219) or 30 μM LPA supplemented with 10 μM Blebbistatin (+) (n = 167), 10 μM Blebbistatin (−) (n = 143), 10 μM Y-27632 (n = 149), 100 nM Latrunculin A (LatA, n = 134), and 500 nM Jasplakinolide (Jasplak) (n = 83). (D) Recruitment of myosin II to the cortex of progenitor cells over time after application of 10 μM LPA by a micropipette. Data (black, mean ± SEM) and sigmoid fit function (red) with half time t_1/2_ = 20 s (n = 30). (E) Myosin II localization in progenitor cells cultured in DMEM-F12 medium alone (Ctrl), 1 μM or 10 μM LPA. Arrows mark blebs (yellow) and the cortex-depleted protrusion front of stable-bleb cells (red). (F) Boxplot of relative cortical myosin II fluorescence intensities for increasing LPA levels in unpolarized progenitor cells (each n = 120). (G) Cortical enrichment of Myl12.1-eGFP (n = 60), Lifeact-GFP (n = 60), and GPI-RFP (n = 33) in non-polarized progenitor cells treated with 10 μM LPA. Cortical fluorescence intensity values I_LPA_ are normalized to reference values without LPA stimulation I_Ctrl_. (H) Relative bleb sizes for increasing LPA levels (each n = 120). (I and J) Relative bleb sizes (I) and relative cortical myosin II fluorescence intensities (J) under non-confined and confined conditions. (K) Exemplary fluorescence images of non-polarized and polarized cells expressing Myl12.1 in non-confined and confined conditions. (L) BF images of mesodermal progenitors cultured in non-confined and confined environments (top) and quantification of cellular protrusions (bottom). (M) Time-lapse BF and fluorescence images of Myl12.1-eGFP localization in mesodermal progenitor cells cultured on a 2D substrate coated with Fibronectin upon LPA stimulation. n.s., not significant, ^∗∗∗^p < 0.001. Boxplots show single cell data points (black) and median (red). All cells were obtained from embryos at sphere stage, despite (B), and cultured in suspension on a passivated substrate despite (L and M). n, number of cells. All scale bars represent 20 μm. See also Figure S1, Movies S2 and S3, and Extended Experimental Procedures.

**Figure S1:**
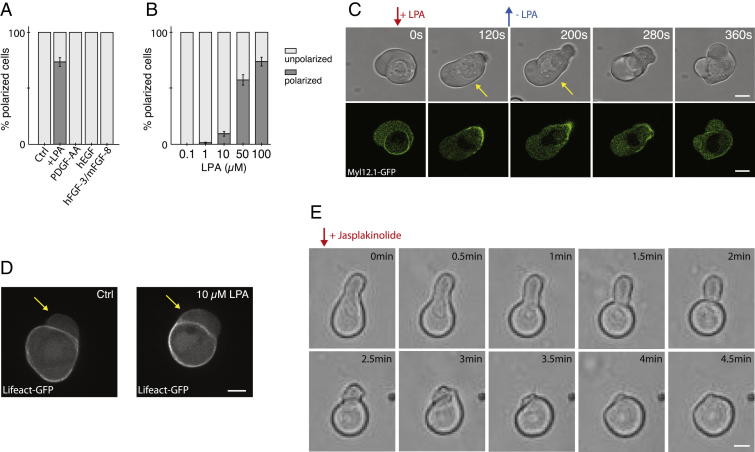
LPA Stimulates Stable-Bleb Cell Transformation In Vitro, Related to Figure 2 (A) Percentage of polarized stable-bleb cells cultured in suspension in the presence of 100 μM LPA for 10 min (n = 219) or different serum growth factors hEGF (n = 143), mFGF-8b+rhFGF-3 (n = 121), and PDGF-AA (n = 162) at a concentration of 100 ng/μl for 30 min. (B) Percentage of polarized stable-bleb cells cultured in suspension at varying levels of LPA for 10 min. (C) Bright-field and fluorescence time-lapse images of Myl12.1-eGFP (myosin II) localization during reversible polarization of progenitor cells obtained from *Tg(actβ1:myl12.1eGFP)* transgenic embryos. Red arrow denotes local application of 100 μM LPA by a micropipette. Blue arrow marks removal of micropipette. Yellow arrows indicate polarized stable-bleb cells. (D) Lifeact-GFP localization in progenitor cells obtained from *Tg(actβ1:lifeact-GFP*) embryos and cultured in DMEM-F12 medium alone (Ctrl, top) and in the presence of 10 μM LPA (bottom). Yellow arrows mark blebs. (E) Bright-field time-lapse images of a polarized progenitor cell cultured in suspension in the presence of 30 μM LPA. Red arrow marks application of 500 nM Jasplakinolide to the culture medium. All cells were obtained from embryos at sphere stage (4 hpf). All scale bars, 10 μm.

To investigate the molecular basis underlying this transformation, we first asked which serum components are involved. While adding serum growth factors, such as fibroblast growth factor (FGF), platelet-derived growth factor (PDGF), or epidermal growth factor (EGF), to the culture medium was not sufficient to induce stable-bleb cells (Figure S1A), we observed a fast and robust transformation when lysophosphatidic acid (LPA) was added (Figures 2A and S1B), a serum phospholipid known to activate cortical contractility via the Rho/Rock pathway (Mills and Moolenaar, 2003).

Polarization was stable in the presence of LPA, but cells switched rapidly back into their original blebbing behavior upon dilution of LPA from the culture medium, indicating that LPA-mediated transformation of progenitors into stable-bleb cells is a reversible process (Figure S1C). Furthermore, cell polarization was observed in suspension at homogenous levels of LPA (Movie S2) suggesting that cell transformation occurs in the absence of extrinsic gradients of biochemical or environmental polarity cues and arguing in favor of a cell intrinsic stochastic polarization mechanism induced by LPA.

Surprisingly, transformation into stable-bleb cells could be triggered in progenitor cells from blastula and gastrula stages irrespective of their primary cell fate (Figure 2B) as determined by dissociating cells from *Tg*(*mezzo:eGFP*) transgenic embryos expressing eGFP in mesendodermal progenitor cells. We thus reasoned that the motile switch occurred independently of a cell-type-specific genetic module, but might rather be associated with a generic polarization mechanism retrievable in these cells.

Given that LPA is known to activate cortical contractility via the Rho/Rock pathway, we hypothesized that a mechanical polarization mechanism of the cell cortex could trigger the transformation of progenitor cells into stable-bleb cells (Carvalho et al., 2013). To test this hypothesis, we treated stable-bleb cells with the Rho kinase inhibitor Y-27632 or the myosin-II inhibitor Blebbistatin (Figure 2C). Treated cells lost their characteristic polarization, supporting a critical role for Rho/Rock-mediated cortical contractility in driving stable-bleb cell transformation.

To address the effect of LPA stimulation on stable-bleb cell polarization, we monitored the distribution of cortical markers in the presence of different LPA concentrations. Myosin II rapidly redistributed to the cell cortex upon LPA stimulation (t_1/2_ ∼20 s, Figure 2D), and rising LPA concentrations were associated with increasing cortical accumulations of myosin II, eventually saturating at a threshold level at which progenitors transformed into stable-bleb cells (Figures 2E and 2F). Importantly, the accumulation of myosin II at the cortex was up to seven times larger than the accumulation of actin, indicating that myosin II-mediated cortical contractility was specifically elevated upon LPA stimulation (Figures 2G and S1D). Furthermore, bleb sizes in dissociated progenitor cells closely scaled with the concentrations of LPA in the medium (Figures 2E and 2H), suggesting that LPA upregulates cortical contractility and enhances bleb expansion by increasing intracellular pressure.

Interestingly, we observed that strong spatial confinement of progenitor cells in serum-free medium also led to elevated levels of myosin II accumulation at the cortex, larger blebs, and stochastic transformation into stable-bleb cells (Figures 2I–2K). In mesenchymal cells, this transformation was typically preceded by an increase in the formation of cellular blebs at the expense of lamellipodia and filopodia (Figure 2L). Together, these results imply that increasing levels of myosin-II mediated cortical contractility by either biochemical or physical stimuli can trigger a rapid and reversible amoeboid transformation of non-polarized and mesenchymal progenitors (Figure 2M; Movie S3) that is accompanied with extensive remodeling of cortical architecture and myosin II localization in those cells. Notably, a similar mesenchymal-to-amoeboid transition (MAT) has been observed in various culture cell types upon compression (see Liu et al. [2015] in this issue of *Cell*), suggesting that contractility-induced MAT is a universal mechanism retrievable in various primary and culture cell types.

### A Contraction-Based Polarization Mechanism Drives Stable-Bleb Cell Transformation

**Figure 3:**
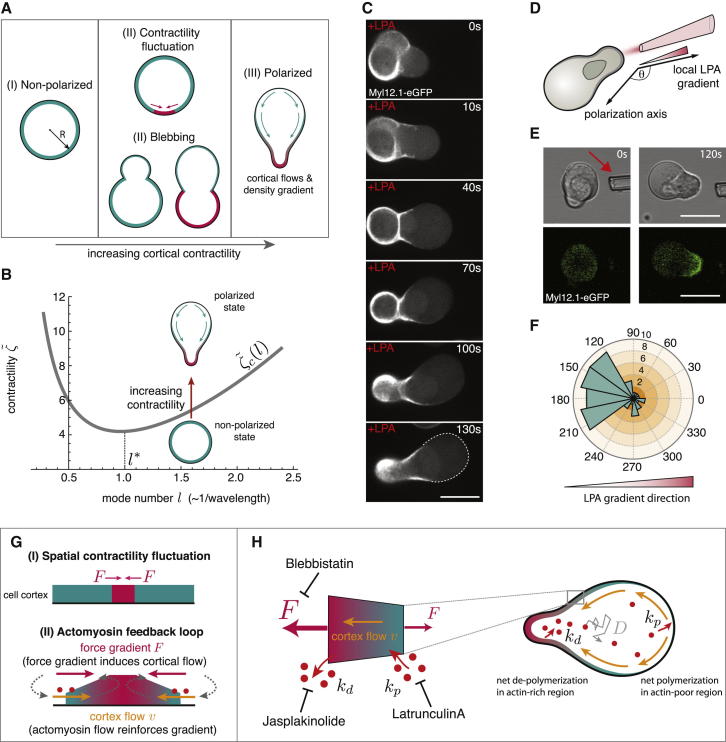
Cortical Contractility Fluctuations Drive Polarization to Stable-Bleb Cells (A) Sketch of polarization from a non-polarized state (left) to a polarized stable-bleb state (right). The cell cortex is shown in green and highly contractile cortex areas in red. Red arrows denote a cortical force imbalance due to local fluctuations in cortical contractility. Blebs induce spatial inhomogeneities in cortical contractility of increasing strength for larger bleb sizes. Green arrows indicate cortical flows. (B) Stability diagram illustrating the switch from a non-polarized to a polarized state. The curve represents the values of cortical contractility ζ, depending on the density fluctuation mode number *l*. The mode number, *l*, is inversely proportional to the wavelength (in units of initial cell radius) of the fluctuation. (C) Myosin II localization in a transforming progenitor cell cultured in suspension with 30 μM LPA. Scale bar represents 20 μm. (D) Sketch of the local LPA application experiment. (E) BF time-lapse images and Myl12.1-eGFP localization during progenitor cell polarization in a local 100 μM LPA diffusion gradient set by a micropipette (red arrow). (F) Histogram of measured cell polarization orientations (n = 32). (G and H) Schematic illustration of the positive feedback loop between cortical actomyosin density gradients and rearward cortical flows for the maintenance of stable-bleb cell polarity. Local fluctuations in cortical contractility induce a flow toward the contractile region along with mass transport of actin and myosin (red dots) that reinforces the initial instability (G). To maintain a continuous cortical flow in polarized cells actin turnover (with rate constants *k*_*p*_ and *k*_*d*_) and diffusion of free actin and myosin (with diffusion constant *D*) is required (H). Polarization is lost upon pharmacological inhibition of myosin II contractility (by Blebbistatin), actin polymerization (by Latrunculin-A) and actin de-polymerization (by Jasplakinolide). See also Figure S2, Movies S2 and S4, and Extended Experimental Procedures.

**Figure S2:**
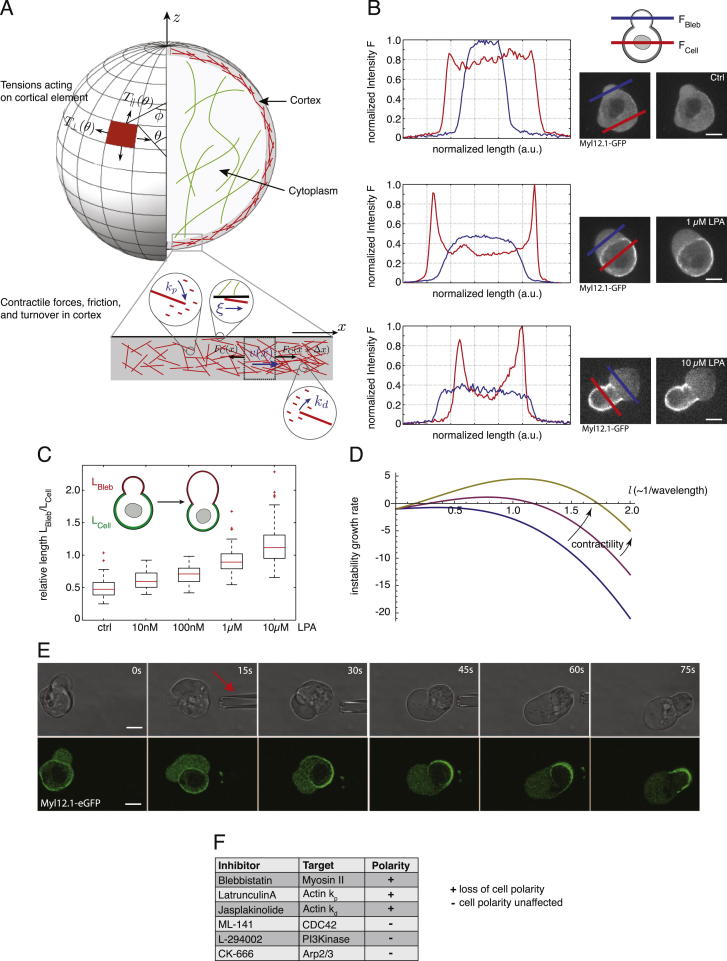
Cortical Contractility Fluctuations Drive Stable-Bleb Cell Transformation, Related to Figure 3 (A) Illustration of contractility-driven cortical instability in a non-polarized cell. In an element of cortex (shown as zoom in), a gradient in actin filament density (filaments are represented by red sticks) results in a net contractile force to the right; this results in a net flow, which amplifies the accumulation of actin on the right. The instability is opposed by actin turnover (given by polymerization and depolymerization rates, *k*_*p*_ and *k*_*d*_), osmotic effects (i.e., steric repulsion between filaments), friction (with coefficient ξ), and actin viscosity. During cortical instability and in the polarized state of the cell, the cortical tension is inhomogeneous, and the tension component parallel to the polarization axis (*T*_∥_) is generally different from the perpendicular component (*T*_⊥_). (B) Myl12.1-eGFP (myosin II) localization in progenitor cells cultured in DMEM-F12 medium alone (ctrl, top) and in the presence of 1 μM LPA (middle) or 10 μM LPA (bottom). Corresponding fluorescence intensity profiles were measured across the cell body (red lines) and bleb protrusions (blue lines), indicating a differential contractility between cell cortex and bleb cortex that increases for higher LPA concentrations. (C) Relative bleb sizes given as the ratio of bleb contour length L_Bleb_ to cell contour length L_Cell_ obtained from fluorescence cross-sectional images of progenitor cells as shown in (B) at varying amounts of LPA (n = 60 for all conditions). (D) Growth rate of cortical instability versus density fluctuation mode number *l* (∼1/wavelength). The growth rate, *λ*_*l*_ in units $R^{4}\xi /\rho_{0}^{2}\gamma$, is shown for three values of cortical contractility, given by the parameter $\tilde{\zeta }=R^{2}/\rho_{0}\gamma \;\left(\zeta -\beta \rho_{0}^{3}\right)$: $\tilde{\zeta }=1$, $\tilde{\zeta }=3$, $\tilde{\zeta }=5$. Other parameter values are *η*/*ξR*^2^ = 0.1, $\zeta^{\prime }\beta_{2}/\xi R^{2}\chi =0,$ and $\left(R^{4}\xi /\rho_{0}^{2}\gamma \right)k_{d}=1$. The curves indicate that, due to the competing effects of contractility and density gradient energy penalties, there is, in general, only a particular window of fluctuation wavelengths that are unstable. (E) Bright-field (top) and fluorescence time-lapse images of Myl12.1-eGFP (myosin II) localization during progenitor cell polarization in a local 100 μM LPA diffusion gradient set by a micropipette (red arrow). (F) Table of pharmacological inhibitors summarizing their molecular targets and effects on stable-bleb cell polarization. Myl12.1-eGFP expressing cells in (B) and (E) were obtained from *Tg(actβ1:myl12.1eGFP)* transgenic embryos at sphere stage (4 hpf). Number of cells is given by n. All scale bars, 10 μm.

Based on these experimental results, we hypothesized that rapid changes in cortical architecture by increasing levels of cortical contractility mediate a cell intrinsic transformation into stable-bleb cells. To address this hypothesis, we developed a theoretical description of the polarization process by modeling cortex structures and associated cell shapes as a function of cortical contractility (see Extended Experimental Procedures). In this model, the cell cortex was treated as a thin spherical shell of compressible active gel (Callan-Jones and Voituriez, 2013; Hawkins et al., 2011) (Figure S2A). Assuming homogenous bulk polymerization and depolymerization rates, we precluded a predetermined symmetry break of the cortical layer. Instead, we treated blebbing events in unpolarized progenitor cells as random spatial fluctuations in cortical contractility (Figure 3A), an assumption supported by measurements of the spatial extent (bleb size) and amplitude of cortical instabilities (myosin II enrichment) during cell blebbing at increasing LPA levels (Figures S2B and S2C). Notably, our model predicted that above a threshold level of cortical contractility these local instabilities would not relax but be amplified, eventually transforming an initially homogenous cortex layer into a polarized steady state characterized by a stable cortical density gradient along with retrograde cortical flows (Figures 3B and S2D).

Indeed, time-lapse imaging of myosin II localization during cell polarization in LPA stimulated progenitor cells confirmed a rapid myosin II re-localization to the cortex and strongly increasing bleb sizes prior to polarization, consistent with elevated levels of cortical contractility fluctuations and an increasing imbalance in contractile forces between the bleb and cellular cortical network (Figure 3C; Movie S4). Stabilization of blebs was initiated by the appearance of directional cortical flows from the weak actomyosin-network assembling underneath the bleb toward the cell cortex, which resulted in a failure of bleb retraction and ultimately led to stable cell polarization (Movie S4). To test whether ectopically increased levels of contractility can trigger cell polarization, we generated a localized LPA diffusion gradient by a micropipette (Figure 3D). Progenitor cells in the presence of this LPA gradient rapidly transformed into stable-bleb cells with their contractile back oriented toward the source of LPA (Figures 3E, 3F, and S2E). This suggests that external gradients of LPA can trigger directional cell polarization by inducing an asymmetric contraction of the cortical cytoskeleton in agreement with theoretical modeling.

**Figure 4:**
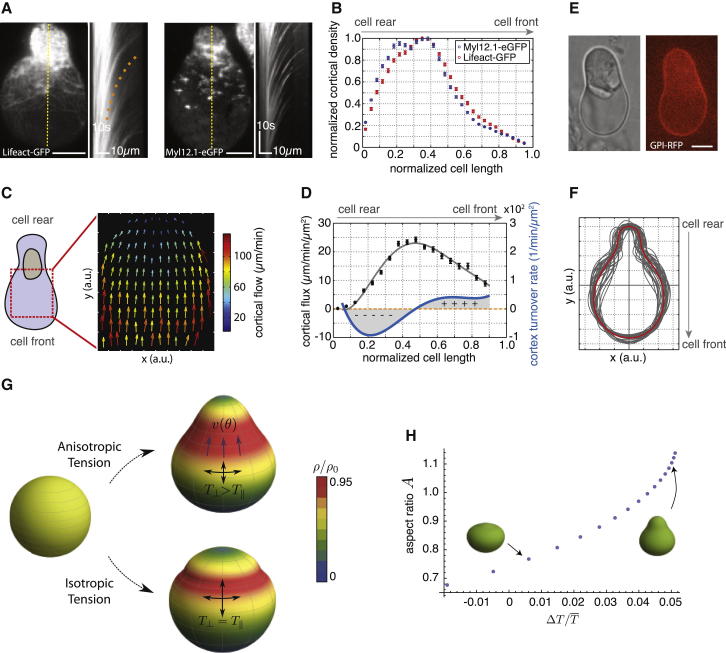
Cortical Architecture Determines Cell Shape of Stable-Bleb Cells In Vitro (A) TIRFM image showing Lifeact-GFP (left) and myosin II localization (right) in isolated stable-bleb cells with corresponding kymograph data along yellow lines. Orange dotted line indicates the cortical flow profile. (B) Average actin and myosin II density profiles obtained from culture conditions in (A) (n = 30). (C) Average 2D cortical flow map in the spherical protrusion front of stable-bleb cells (n = 3). The red dashed square highlights the analyzed cell area. (D) Cortical flux data (black, mean ± SEM) calculated from average cortical density (B) and average cortical flow profiles (Figure S3C) with polynomial fit (gray) and cortex turnover rate (blue) calculated as the derivative of the cortical flux (n = 30). (E) BF and fluorescence cross-sectional image of an isolated stable-bleb cell expressing GPI-RFP. (F) Average (red) and single cell shapes (gray) of polarized stable-bleb cells (n = 30). (G) Theoretical predictions on polarized cell shapes generated by cortical tension components parallel *T*_*II*_ and perpendicular *T*_⊥_ to the polarization axis. Top: anisotropic tension (*T*_*II*_ < *T*_⊥_). Bottom: isotropic tension (*T*_*II*_ = *T*_⊥_). Color bar represents normalized cortical density distributions. Magenta arrows indicate the direction of cortical flow *v*(*θ*) in polar direction. Parameter values are provided in the Extended Experimental Procedures. (H) Phase diagram of cell shapes with cell aspect ratio *A* as a function of tension anisotropy. Elongated, pear shapes are obtained for higher anisotropy. All cells were cultured on a PEG-coated substrate either with (A–D) or without confinement (E and F). Scale bars represent 10 μm. See also Figure S3 and Extended Experimental Procedures.

**Figure S3:**
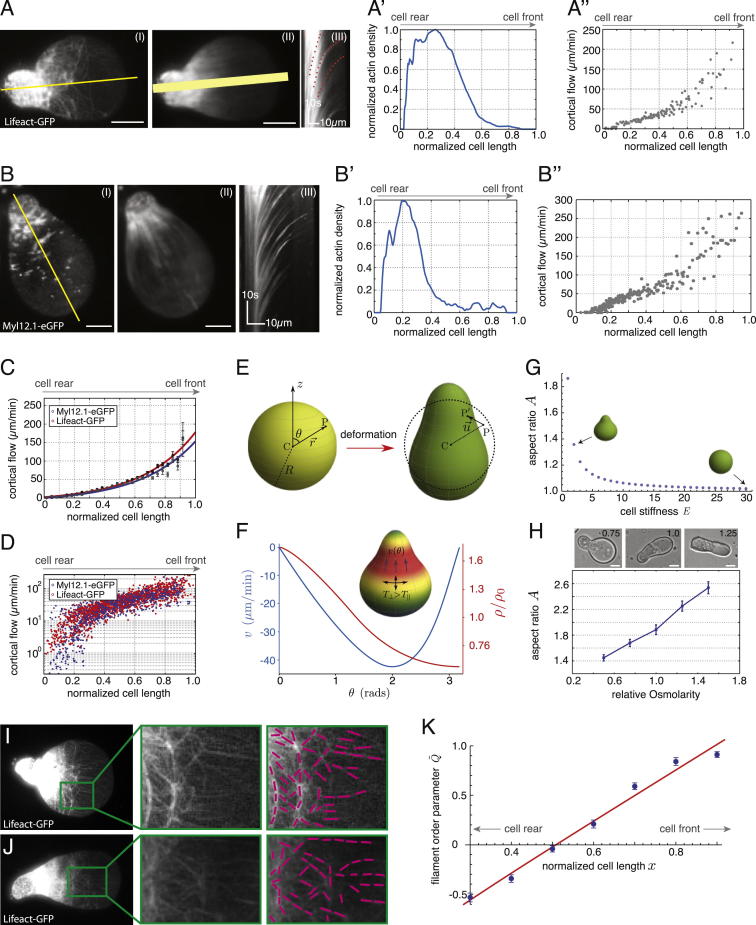
Actomyosin Cortical Flows and Spatial Cortical Density Gradients Determine the Shape of Polarized Stable-Bleb Cells, Related to Figure 4 (A) TIRFM image (I) of a polarized stable-bleb cell obtained from *Tg(actβ1:lifeact-GFP*) transgenic embryos at sphere stage (4 hpf) and cultured in confinement under agarose on a PEG-coated substrate in the presence of 30 μM LPA. (II) Averaged fluorescence image over 50 frames and (III) kymograph data along yellow line in (I). (A′) Single cell density profile along yellow line in (II). Width of yellow line in (II) indicates the lateral line extent used for calculating average density profiles. (A″) Single cell cortical flow profile obtained from kymograph data in (III). (B) TIRFM image (I) of a polarized stable-bleb cell obtained from *Tg(actβ1:myl12.1eGFP)* transgenic embryos at sphere stage (4 hpf) and cultured in confinement under agarose on a PEG-coated substrate in the presence of 30 μM LPA. (II) Average fluorescence image over 50 frames and (III) kymograph along the axis of polarization. (B′ and B″) Single cell density (B′) and cortical flow profile (B″) obtained along yellow line in (II) and kymograph data (III). (C) Average cortical flow profile of data for Myl12.1eGFP (gray, mean ± sem; n = 27) with exponential fit (blue) and Lifeact-GFP (black, mean ± sem; n = 30) with exponential fit (red). (D) Log-Linear plot of single cell cortical flow data obtained from (C). (E) Illustration of cell shape change resulting from cortical tension. The un-deformed state of the cell is assumed to be spherical with radius *R*. An arbitrary point *P* in the cytoplasm gets shifted to *P′* under deformation, by an amount $\vec{u}$. (F) Cortical flow and density profile in the polarized state of the cell. In the steady state, inhomogeneous solutions to *v*(Θ) and *ρ*(Θ) determine the cortical tensions and friction that are exerted on the bulk of the cell, and, as a result, the cell shape in the polarized state (shown in inset). The parameter values for the determination of *v*(Θ) and *ρ*(Θ), and the cell deformation are: $\zeta^{\prime }\beta_{2}/\xi R^{2}\chi =2$, *η*/*ξR*^2^ = 0.1, $\left(R^{4}\xi /\rho_{0}^{2}\gamma \right)k_{d}=1$, *β*_2_ = 1, and $\left(R^{4}\xi /\rho_{0}^{2}\gamma \right)\chi =1.$ The cortical flow is indicated in μm/min (taking *k*_*d*_ = 0.1 *s*^−1^ (Pollard and Borisy, 2003) and *R* = 20 *μm*), while the actin density is expressed relative to the unperturbed value, *ρ*_0_ (which, without loss of generality, we take to be equal to one). (G) Phase diagram of cell shapes showing cell aspect ratio *A* versus cell stiffness *E*. (H) Representative bright-field images of polarized progenitor cells cultured at varying osmotic conditions in the presence of 30 μM LPA with numbers indicating relative medium osmolarities (standard medium 1.0, hypoosmotic 0.75, hyperosmotic 1.25; top) and quantification of cell aspect ratios for varying medium osmolarities (n = 50 cells for all conditions). (I and J) TIRF images of Lifeact-GFP localization in polarized progenitor cells obtained from *Tg(actβ1:lifeact-GFP*) embryos cultured in non-adhesive confinement under agarose in the presence of 30 μM LPA (left). Magnification of green highlighted area (middle) and tracking of filament alignment (right). (K) Quantification of filament alignment order parameter *Q* along the normalized cell length x with theoretical fit function (see Equations S29, S30, and S31). All scale bars, 10 μm.

We next asked how stable-bleb cells maintain their polarity. According to our theoretical model, we expected that cell polarization in stable-bleb cells could potentially be maintained by a positive feedback loop between cortical contractility gradients and the presence of cortical flows (Bois et al., 2011; Hawkins et al., 2011). In this positive feedback, cortical flows reinforce density gradients, in particular rearward localization of myosin II, and thereby reinforce contractility gradients that drive cortical flow toward the contractile region (Figure 3G). High resolution TIRF imaging of cortical actin and myosin II in polarized progenitor cells confirmed the presence of stable cortical density gradients toward the cell rear and revealed a low density actomyosin network in the spherical protrusion front (Figures 4A, 4B, S3A, and S3B). This sparse actomyosin meshwork was reminiscent of a bleb-like membrane blister but, unlike blebs, was accompanied by an unusually fast and continuous cortical actomyosin flow, referred to as cortical flow in the following, with maximal flow speeds up to 150 μm/min in the very cell front (Figures 4A, 4C, S3C, and S3D). Measurement of cortical flows along with cortical density profiles allowed for calculating cortex flux and cortex turnover rate (Figure 4D), indicating net polymerization in the spherical protrusion front and de-polymerization toward the rear. As a continuous rearward cortex flux requires permanent cortex turnover, stable-bleb cell polarization was rapidly lost upon treatment with the G-actin sequestering drug Latrunculin A or Jasplakinolide, a drug that interferes with cortex turnover (Figures 2C and S1E; Movie S2). Moreover, treatment with the myosin II inhibitor Blebbistatin also reversed cell polarization (Figure 2C), indicating that cortical flow in combination with a gradient in contractility is critical for maintaining stable-bleb cell polarization over time. In contrast, inhibition of CDC42 (ML-141) or PI3Kinase (L-294002), previously shown to be required for mesendodermal progenitor cell migration in vivo (Montero et al., 2003), did not affect stable-bleb cell polarization (Figure S2F), supporting the concept that stable-bleb cell motility is unrelated to actin driven protrusion types such as lamellipodia or filopodia. Collectively, our results support a simple mechanical model of stochastic cell polarization based on the amplification of local fluctuations in cortical contractility and a positive feedback mechanism between contractility gradients and continuous cortical flows maintaining polarity in stable-bleb cells (Figure 3H).

To determine how the polarized cortical scaffold in stable-bleb cells connects to their characteristic shape, we expanded our theoretical model to calculate cell shapes based on deformations of the cytoplasm due to compressive and shear stresses at the cytoplasmic surface arising from cortical tension and flow-friction (Behrndt et al., 2012) (Figures S3E and S3F). The cytoplasm was modeled as a viscoelastic medium with residual elastic response at long times (Tinevez et al., 2009), which is the regime of interest for discussing steady-state shapes. While measured actomyosin density gradients serve as a readout of cortical contractility, cortical tension in general is related not only to contractility but also to viscous forces arising from flow (Mayer et al., 2010) and flow-induced filament alignment (Salbreux et al., 2009). Using experimentally determined cortical density profiles we calculated shapes for two different cases: In the first case, we assumed an isotropic tension resulting in flattened, bulbous cell shapes not matching our experimental observations (Figure 4G, bottom). In the second case, we included viscous forces leading to tension reduction along the flow direction. Such tension anisotropy resulted in elongated pear shapes closely mirroring the observed shapes of stable-bleb cells (Figure 4G, top). In support of our theoretical predictions, we observed a preferential alignment of actin filaments perpendicular to the front-to-rear axis of stable-bleb cells, highlighting the presence of anisotropic tension within the cortex (Figures S3I–S3K). Finally, we tested whether stable-bleb cell shapes depend on cortical viscosity and cell stiffness (Figures 4H and S3G) and used osmotic shock experiments to alter intracellular pressure and, consequently, cell stiffness. Consistent with our theoretical predictions, we found that decreasing intracellular pressure resulted in more elongated cell shapes (Figure S3H). Together, our theoretical considerations suggest that the characteristic pear-shape morphology of stable-bleb cells is generated by anisotropic tension in the cell cortex, which acts as a belt-like constriction unit applying compressive forces normal to the axis of polarization.

### Retrograde Cortical Flows Drive Stable-Bleb Cell Migration

**Figure 5:**
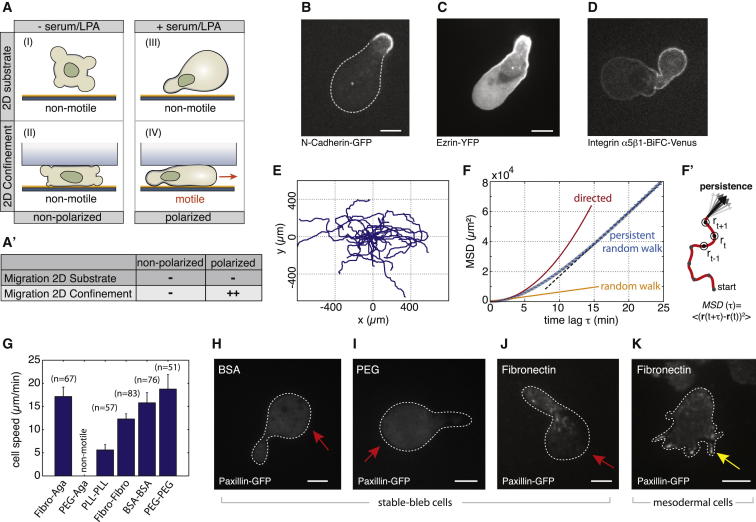
Migration Characteristics of Stable-Bleb Cells In Vitro (A) Illustration of progenitor cells cultured under serum/LPA-free (I, II) or serum/LPA-containing (III, IV) conditions with (II, IV) or without (I, III) confinement. (A′) Table summarizing cell migration characteristics for different culture conditions. (B–D) N-Cadherin-GFP (B), Ezrin-YFP (C), and Integrinα5-Integrinβ1-BiFc-Venus (D) localization in polarized stable-bleb cells. (E) Representative tracks of migrating stable-bleb cells. (F) Mean-square displacement (MSD) analysis data (blue, n = 67) with fit to a persistent random walk model (gray) and compared to random (∼t, orange) and directed migration (∼t^2^, red). Black dashed line highlights random migration on timescales above the persistence time P_t_ ∼10 min (persistence length ≈150 μm). (F′) Sketch of MSD time point analysis. Arrows denote variations in the direction of movement affecting persistence of cell movement. (G) Average instantaneous stable-bleb cell migration speeds in confinement between glass-agarose or glass-glass assays for varying substrate coatings as indicated. (H–J) Localization of Paxillin-GFP for stable-bleb cells on BSA- (H), PEG- (I), and Fibronectin-coated (J) glass substrates in confinement. (K) Paxillin-GFP localization for mesodermal progenitors cultured on 2D planar substrates coated with Fibronectin. n denotes number of cells. White dashed lines in (H)–(K) outline cell contact areas. All scale bars represent 10 μm. See also Extended Experimental Procedures.

To study the migratory behavior of polarized stable-bleb cells, we monitored their motility in different environments (Figure 5A). Stable-bleb cells only weakly adhered to and failed to migrate on 2D adhesive substrates coated with ECM components, such as Laminin and Fibronectin. Fluorescence imaging of the subcellular distribution of molecules associated with coupling the cortex to the surrounding substrate, such as N-Cadherin, Ezrin, and Integrin revealed a highly asymmetric accumulation toward the cell rear, while the bleb-like protrusion front was largely devoid of those molecules (Figures 5B and 5C). This indicates only weak adhesive forces in the front that are insufficient for forward locomotion on 2D substrates. To increase frictional coupling of migrating cells, we thus created a planar 2D confinement by placing cells between a coated glass substrate and an agarose layer. We found that stable-bleb cells plated on Fibronectin-coated substrates under confinement displayed exceptionally fast and directional movements with average cell speed <*v*_*cell*_> = 17.1 ± 0.6 μm/min (n = 67 cells) and persistence length of approximately four cell diameters (Figures 5E and 5F), best described by a persistent random walk model over long timescales (Figure 5F).

**Figure S4:**
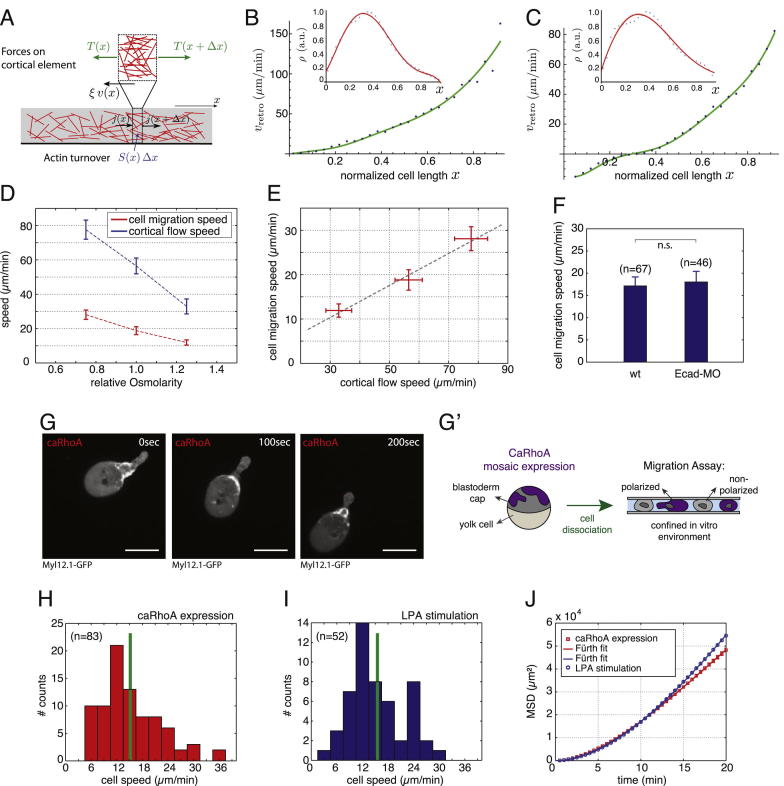
Cortical Flows Drive Stable-Bleb Cell Migration via Frictional Coupling to the Environment, Related to Figure 6 (A) Schematic illustration of a one-dimensional cortex layer with an actin filament gradient (red sticks) flowing with speed *v*(*x*). The forces per unit length acting on a cortex element of length *dx* are the tensions *T*(*x*), *T*(*x* + *dx*), and the frictional force *ξv*(*x*)*dx*. (B) Retrograde flow speed versus distance along flow direction for non-motile cells on PEG (slipping conditions). Symbols indicate the experimental data for average cortical flow speed *v*_*retro*_ obtained from Figure 6G; inset shows cortical density data with fit (red) to a fifth-order polynomial in *x*. From the fit to *v*_*retro*_ (green) we obtain the friction coefficient ξ_PEG_ ≃ 10^8^ Pa·s/m. (C) Retrograde flow speed versus distance along flow direction for motile cells. Symbols indicate the experimental data for *v*_*retro*_ obtained from Figure 6H; inset shows cortical density data and fit (red) to a fifth-order polynomial in *x*. From the fit to *v*_*retro*_ (green) we obtain the friction coefficient ξ_running_ ≃ 2.8 × 10^8^ Pa·s/m. (D) Average cell migration speed (red) and average cortical flow speed (blue) measured at x = 0.7 normalized cell length (*x* = 0, cell rear; x = 1, cell front) for varying relative medium osmolarities (standard medium 1.0, hypoosmotic 0.75, hyperosmotic 1.25). (E) Correlation plot of cell migration speed versus cortical flow velocity from data obtained in (D). (F) Mean instantaneous cell migration speed of polarized stable-bleb cells obtained from wild-type embryos (wt) or wild-type embryos injected with E-Cadherin *morpholinos* (Ecad-MO) cultured in confinement in the presence of 30 μM LPA. (G) Fluorescence time-lapse images of Myl12.1-eGFP (myosin II) localization in a polarized progenitor cell expressing a constitutively active version of rhoA (caRhoA) obtained from wild-type embryos with mosaic expression of caRhoA. (G′) Schematic illustration of experimental configuration in (G). Polarized and non-polarized progenitor cells are obtained by dissociation of caRhoA mosaic embryos and cultured in confinement on non-coated glass substrates in the absence of serum or growth factors such as LPA. (H) Mean instantaneous cell migration speed of polarized caRhoA expressing stable-bleb cells cultured as described in (G′). (I) Mean instantaneous cell migration speed of polarized stable-bleb cells obtained from wild-type embryos and cultured in conditions similar to (H) but in the presence of 30 μM LPA. (J) Mean square displacement (MSD) analysis of cell trajectories obtained from data in (H) and (I). All cells were obtained from embryos at sphere stage (4 hpf). All scale bars, 20 μm.

To test the role of specific versus unspecific adhesion, we monitored stable-bleb cell migration in various adhesive and non-adhesive environments using a modified assay that consists of two parallel glass slides coated with either Fibronectin, poly-L-lysine (PLL), BSA, or polyethyleneglycol (PEG) separated by 15-μm beads. Surprisingly, stable-bleb cells were highly motile under all conditions with their migration efficacy increasing in non-adhesive environments (Figure 5G). To monitor the formation of adhesive foci in stable-bleb cells during migration, we further imaged Paxillin-GFP, an adaptor protein known to be involved in Integrin-mediated force transduction and focal adhesion assembly in motile cells (Turner, 2000). When stable-bleb cells were placed between Fibronectin-coated surfaces, Paxillin-GFP localized in distinct spots at cell-substrate contacts similar to mesenchymal mesodermal cells cultured on 2D planar Fibronectin-coated substrates, while Paxillin-GFP appeared homogeneously distributed on non-adhesive BSA and PEG-coated surfaces (Figures 5H–5K). This suggests that stable-bleb cells are able to generate both Integrin-dependent and independent surface attachments during migration. To further investigate the role of cell-substrate versus cell-cell adhesion, we also reduced E-Cadherin expression in stable-bleb cells by injecting antisense *morpholino* oligonucleotides (MOs) and found that E-Cadherin is dispensable for cell migration in vitro irrespective of the substrate coating (Figure S4F). Together, these data support that stable-bleb cells are able to generate both specific and unspecific traction forces during migration, highlighting their versatile migration competence in various adhesive and non-adhesive environments.

**Figure 6:**
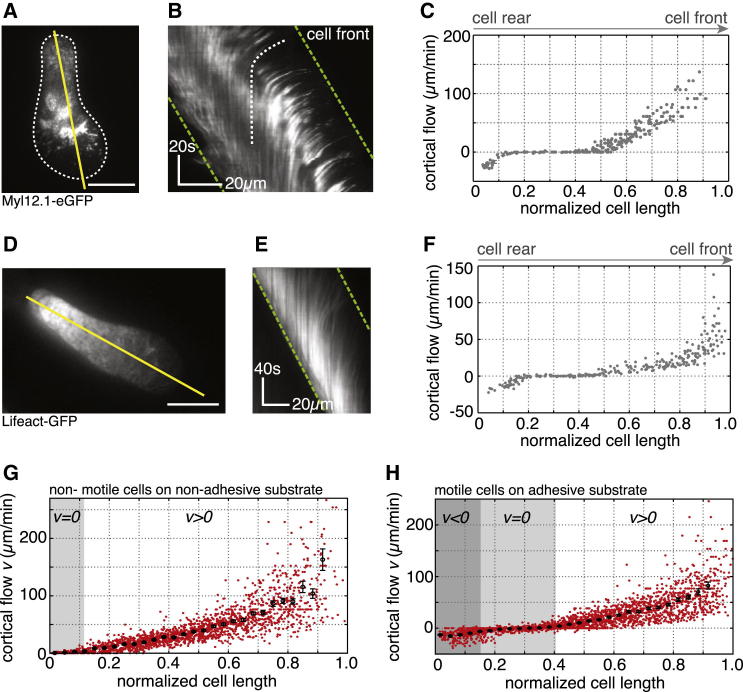
Frictional Coupling of Retrograde Cortical Flows Drives Stable-Bleb Migration (A and D) TIRFM images of (A) Myl12.1-eGFP and (D) Lifeact-GFP localization in motile stable-bleb cells. White dashed line marks the cell-to-substrate contact area. Scale bars represent 10 μm. (B and E) Kymograph data along the direction of movement indicated by yellow lines in (A) and (D). Green dashed lines outline cell front and rear advancement. White dashed line highlights the rearward flow of a representative fluorescent patch. (C and F) Retrograde cortical flow velocity data in single stable-bleb cells obtained from kymograph data in (B) and (E) with v_Cell_ = 24.1 μm/min (C) and v_Cell_ = 18.2 μm/min (F). (G and H) Average (black) and single cell local flow velocity data (red) of migrating stable-bleb cells (H) (n = 15) with average cell speed v_Cell_ = 19.2 ± 2.4 μm/min and of non-motile stable-bleb cells (G) (n = 30) cultured in confinement under agarose on a non-adhesive PEG-coated substrate (v_Cell_ = 0). Shaded areas denote regions with zero (*v* = 0, light gray) or anterograde (*v* < 0, dark gray) cortical flow. Cortical flows are shown in the laboratory frame of reference. All cells were cultured in the presence of 30 μM LPA in confinement under agarose on Fibronectin-coated substrates despite data in (G). n, number of cells. Error bars represent mean ± SEM. See also Figure S4, Movie S5, and Extended Experimental Procedures.

To understand this efficient and persistent motile behavior, we asked how stable-bleb cells move in 2D confined environments. Depletion of cortical elements in the cell front (Figure 4A) and the diffuse zone of actin net polymerization in the spherical front (Figure 4D), exclude a classical polymerization-driven locomotion strategy, where polymerizing actin filaments push the leading edge plasma membrane forward. However, the presence of fast retrograde cortical flows led us to hypothesize that coupling of the flowing actin network to the surrounding substrate powers stable-bleb cell migration under 2D confinement. To test this hypothesis, we performed high-resolution live cell imaging of retrograde cortical flows in stable-bleb cells in confinement under agarose. Motile stable-bleb cells on adhesive Fibronectin-coated substrates showed a rapid decay of flow velocities toward the cell rear with a pronounced zone of zero flow speed (Figures 6A–6F and 6H; Movie S5). In contrast, non-motile stable-bleb cells that were placed on passivated PEG-coated substrates revealed considerably different retrograde flow profiles with similar maximal flow velocities to motile cells in the cell front, but a slower decay of flow speeds to the cell rear (Figures 6G and S3D). Using a 1D fluid description of the cortex, we estimated from those cortical flow profiles a higher frictional coefficient ξ on adhesive in comparison to non-adhesive surfaces (ξ_adhesive_ ≃ 2.8 × 10^8^ Pa × s/m versus ξ_non-adhesive_ ≃ 1 × 10^8^ Pa × s/m; Figures S4A–S4C). Furthermore, osmotic shock experiments revealed that cortical flows are progressively decreased for lower levels of intracellular pressure and that these changes in cortical flow velocity strongly correlate with cell migration speed (Figures S4D and S4E). These findings support the notion that coupling of the cortex to the surrounding substrate leads to a reduction of cortical flow in motile cells, providing the necessary forward traction force required for cell movement.

### Evidence for Stable-Bleb Cell Migration In Vivo

**Figure 7:**
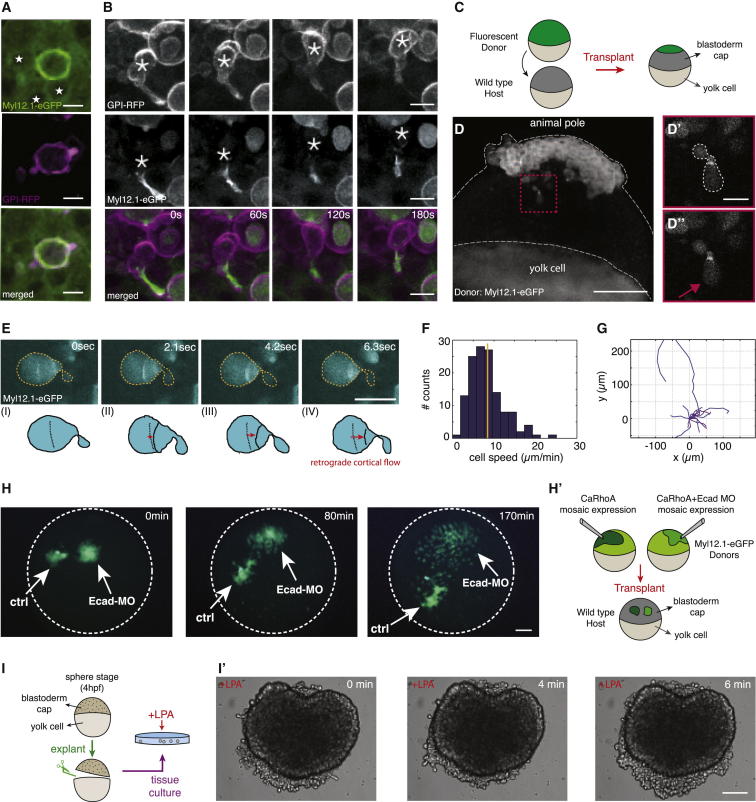
Identification and Characterization of Stable-Bleb Cells In Vivo (A) Myl12.1-eGFP (green) and GPI-RFP (magenta) localization in non-polarized embryonic cells observed in a *Tg*(*actβ1:myl12.1eGFP*) transgenic embryo at sphere stage expressing caRhoA and GPI-RFP in a mosaic pattern. Asterisks denote cell nuclei in the vicinity of a caRhoA expressing cell highlighting cells with reduced cortical accumulation of myosin II compared to caRhoA expressing cells. Scale bars, 10 μm. (B) Fluorescence time-lapse series of a stable-bleb like cell in a *Tg*(*actβ1:myl12.1eGFP*) transgenic embryo at sphere stage expressing caRhoA and GPI-RFP in a mosaic pattern. Scale bars represent 10 μm. (C) Illustration of the transplantation experiment in (D). (D) Transplantation of cells from a *Tg*(*actβ1:myl12.1eGFP*) transgenic donor to a wild-type host embryo at sphere stage. A stable-bleb-like cell emerging from the transplanted tissue is highlighted in the red box. Scale bar represents 100 μm. (D′ and D″) Magnified views on transplanted stable-bleb-like cells observed in (D). Dashed white line marks cell borders. Red arrow indicates the spherical protrusion front. Scale bar represents 20 μm. (E) Myl12.1-eGFP localization and cortical flow in a stable-bleb cell obtained from transplantation of *Tg*(*actβ1:myl12.1eGFP*) transgenic donor cells into a wild-type host. Red arrows point to the direction of cortical flow. Dashed yellow lines outline cell borders. Scale bar represents 25 μm. (F and G) Representative tracks (F) and instantaneous migration speeds (G) of stable-bleb cells in vivo (n = 15 cells) with average velocity (<v_cell_> = 8.5 ± 0.3 μm/min; yellow line). (H) Time-lapse fluorescence images of a cell transplantation between Myl12.1-eGFP donor embryos expressing caRhoA (Ctrl) or caRhoA+E-Cadherin morpholino (Ecad-MO) and a wild-type host at dome stage. Dashed white lines outline the embryo margin. 3Scale bar represents 100 μm. (H′) Schematic illustration of the transplantation experiment in (H). (I) Sketch of tissue explant preparation and culturing. (I′) BF time-lapse images of a blastoderm tissue explant obtained from wild-type embryos at sphere stage and cultured with 30 μM LPA. Scale bar represents 100 μm. See also Figure S5, Movies S6, S7, and S8, and Extended Experimental Procedures.

**Figure S5:**
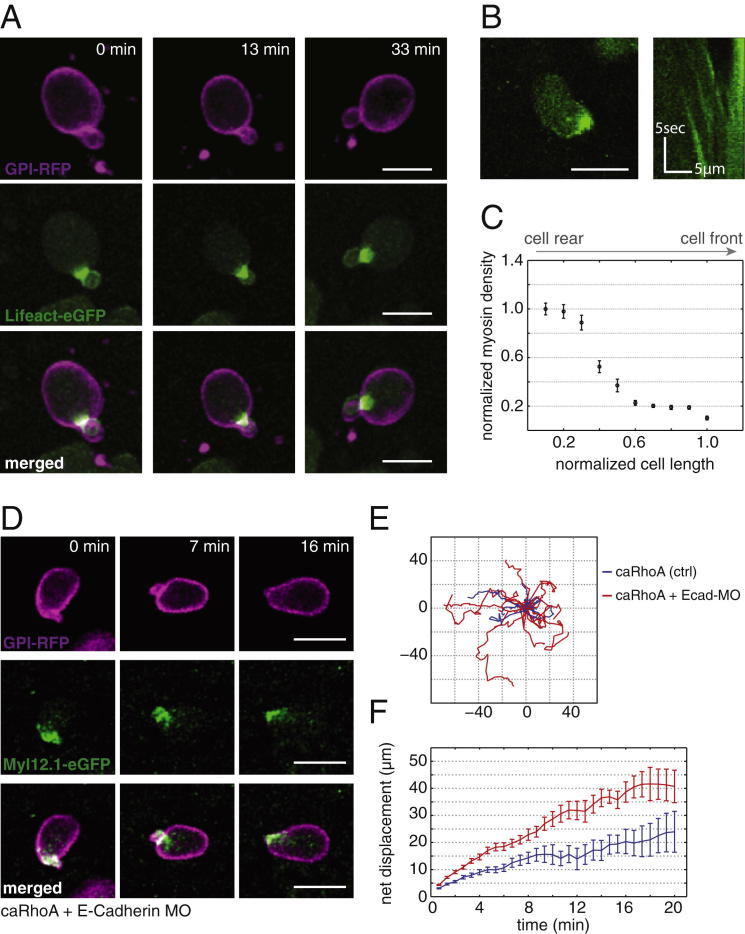
Characterization of Motile caRhoA-Expressing Cells In Vivo, Related to Figure 7 (A) Fluorescence time-lapse images of a single polarized stable-bleb cell transplanted from a transgenic *Tg(actβ1:lifeact-GFP*) donor with mosaic expression of caRhoA plus GPI-RFP into a wild-type host. (B) Localization of Myl12.1-eGFP (myosin II) in a single polarized progenitor cell transplanted from a transgenic *Tg(actβ1:myl12.1eGFP)* donor with mosaic expression of caRhoA plus GPI-RFP into a wild-type host (left) with kymograph data along cell margin (right). (C) Quantification of Myl12.1-eGFP (myosin II) localization in polarized stable-bleb cells along normalized cell length in vivo (n = 4 cells). (D) Fluorescence time-lapse images of a single polarized stable-bleb cell transplanted from a transgenic *Tg(actβ1:myl12.1eGFP)* donor with mosaic expression of a constitutively active version of rhoA (caRhoA) plus GPI-RFP plus *e-Cadherin morpholinos* (MO) into a wild-type host. (E) Exemplary 2D projected in vivo tracks of polarized motile cells expressing either caRhoA (blue) or caRhoA plus *e-Cadherin* MO (caRhoA+Ecad-MO, red) obtained from transplantation experiments as described in (D). (F) Quantification of average single cell net displacement over time for polarized motile cells expressing either caRhoA (blue) or caRhoA plus *e-Cadherin* MO (caRhoA+Ecad-MO, red). All scale bars, 20 μm.

To study the motile switch and migration characteristics of stable-bleb cells in vivo, we first aimed to ectopically transform cells in the early zebrafish blastula by elevating their cortical contractility. To this end, we expressed a constitutively active version of RhoA (caRhoA) (Takesono et al., 2012) in single progenitor cells and performed two-photon imaging of their morphology and migration properties in the gastrulating embryo. CaRhoA expressing cells in vivo showed high blebbing rates and pronounced cortical myosin accumulations similar to LPA-stimulated progenitor cells in vitro (Figure 7A). Moreover, caRhoA-expressing cells frequently transformed into motile cells closely resembling stable-bleb cells in vitro displaying a pear-shape, pronounced cortical actin and myosin II gradients and rearward cortical flows of similar magnitude as found in vitro (v_cortex_ ∼30 μm/min; Figures 7B and S5A–S5C; Movie S6). To further test whether caRhoA expressing progenitor cells in vivo indeed resemble stable-bleb cells in vitro, we transferred them into culture and found that they exhibited a morphology and migration behavior indistinguishable from LPA-induced stable-bleb cells in vitro (Figures S4G–S4J). This suggests that upregulation of cortical contractility through expression of caRhoA is sufficient to transform progenitor cells into motile stable-bleb cells in vivo.

To unravel the role of cell-cell adhesion for adhesive force coupling during stable-bleb cell motility in vivo, we downregulated E-Cadherin expression in caRhoA expressing embryos by injecting specific *e-Cadherin* MOs. Upon transplantation of those cells into a wild-type host we found that E-Cadherin was dispensable for stable-bleb migration in vivo and even led to an increase of cell dispersal compared to caRhoA expressing cells with normal levels of E-Cadherin (Figures 7H and S5D–S5F).

We next sought to address if stable-bleb cells also emerge in the endogenous context of embryonic development. Time-lapse movies of cell movements within the gastrulating embryo occasionally revealed single fast migrating amoeboid cells reminiscent of stable-bleb cells (see Movie S7). However, since it was impossible to predict where and when in the gastrulating embryo these cells appear, we reasoned that locally increasing cortical contractility within the gastrulating embryo might trigger stable-bleb cell transformation. Wounding sites have previously been shown to display elevated levels of actomyosin contractility (Sonnemann and Bement, 2011). To test if stable-bleb cells emerge at local wounding sites within the gastrulating embryo, we transplanted fluorescently labeled donor cells into an unlabeled host embryo at sphere stage (4 hr post fertilization [hpf]) (Figures 7C and 7D). Using *Tg*(*actβ1:myl12.1eGFP*) donor embryos, elevated cortical accumulations of myosin II were detected around the wounding area (Movie S8) and, interestingly, labeled donor cells stochastically transformed into stable-bleb like cells with a highly polarized cortical architecture and fast retrograde cortical flows similar to their morphology in vitro (Figure 7E; Movie S8). Transformed cells migrated away from the region of transplantation with speed (v = 8.5 ± 0.3 μm/min) and directionality much higher than any other cells yet described in the zebrafish embryo (Figures 7F and 7G). Together, this indicates that stable-bleb motility can be induced at places of high contractility within the gastrulating embryo, suggesting a mechanism for rapid cell extrusion from highly contractile regions within the embryo. To further test this assumption, we prepared tissue explants from early blastula stage embryos and cultured them in LPA containing medium. We found that marginal cells in tissue explants not only rapidly transformed into stable-bleb cells, as expected for LPA-treated progenitors, but also frequently extruded from the explant surface (Figure 7I). This suggests that stable-bleb cells can overcome the surface tension of those tissue explants and freely disperse into the environment, supporting our notion of stable-bleb cell transformation as an efficient cell extrusion mechanism from contractile tissues.

## Discussion

Here, we describe a stochastic amoeboid motility switch of early zebrafish progenitor cells into stable-bleb cells. Cell morphology and migration characteristics of stable-bleb cells show unique features in comparison to other motile cell types described before. The invariant roundish cortex-depleted front end of stable-bleb cells is clearly different from actin rich protrusions, such as lamellipodia and filopodia typically found in mesenchymal cells and also distinct from contractility-driven bleb protrusions that periodically appear in blebbing cells. We show that stable polarization of transformed progenitor cells is maintained by a positive feedback loop between cortical density gradients and continuous cortical flows, yielding a robust steady-state configuration with a highly polarized cortical architecture. Interestingly, we found that increasing cortical contractility is sufficient to switch progenitor cells from a blebbing or mesenchymal mode to a stably polarized state above a critical threshold level of contractility, and that this transformation is reversible upon lowering myosin II activity below a critical threshold level. This indicates that the level of cortical contractility determines whether cells migrate by forming either transient protrusions, such as blebs and lamellipodia, or one stably polarized front thereby increasing their migration persistence and efficacy. Supported by theoretical modeling of progenitor transformation into stable-bleb cells, it further suggests that this process does not require an elaborate genetic program, but rather occurs as a simple mechanical switch triggered by increased cortical contractility. This generic polarization module can likely be activated via diverse cellular pathways such as microtubule disassembly-dependent activation of the Rho/ROCK pathway (Niggli, 2003) and could thus be a potentially effective polarization mechanism also functional in other cell types. Notably, findings in various culture cell lines suggest that high spatial confinement combined with low substrate adhesion is sufficient to trigger an amoeboid cell transformation strikingly similar to stable-bleb cells (see Liu et al., 2015). Together, these data suggest that the regulation of cortical contractility might serve as a universal switch for transforming cells into an amoeboid cell migration mode characterized by fast and highly directional migration in 3D confinement.

Spatially confined environments promote stable-bleb cell migration by providing compressive forces that increase frictional coupling required for efficient locomotion. Moreover, retrograde cortical flows are sufficient to drive stable-bleb cell migration in both adhesive and non-adhesive confined environments. Similar flow-friction mechanisms have recently been proposed to drive locomotion of various cell types in confinement (Hawkins et al., 2009; Liu et al., 2015), suggesting that force-coupling via retrograde cortical flows might constitute a general mechanism of cell movement especially in low adhesive 3D environments.

During gastrulation cell motility needs to be precisely controlled and spatio-temporally regulated. Recent studies have pointed at important functions of small Rho GTPases in cell migration during zebrafish gastrulation (Matthews et al., 2008; Kardash et al., 2010). Our observation that LPA, a RhoA-activating signal ubiquitously expressed during zebrafish gastrulation (Lee et al., 2008; Yukiura et al., 2011), can transform progenitor cells into stable-bleb cells, reveals a yet unrecognized function of RhoA in the control of cell migration during gastrulation and points at LPA as one potential regulator determining stable-bleb cell induction during gastrulation. However, given the large number of known signals with RhoA activating capacity, a systematic functional analysis of these signals will be needed to dissect the upstream regulation of stable-bleb cell transformation during gastrulation.

As to the function of stable-bleb cells in gastrulation, we observed stable-bleb cell transformation at areas of high cell contractility, such as wounding sites, followed by rapid dispersal of those cells. This suggests that stable-bleb transformation serves as a mechanism for cell extrusion from areas of increased contractility in the embryo. This might help in equilibrating contractility-driven cell density variations, but might also be used as a mean of long-range communication where extruding stable-bleb cells rapidly transmit signals from areas of high contractility to the remainder of the embryo. Our observation of stable-bleb cell extrusion from tissue explants along with observations of a similar migration phenotype in various cancer cell lines (see Liu et al., 2015) also points at the intriguing possibility that stable-bleb cells might be involved in other contexts such as cancer metastasis where cancer cells disseminate from the primary tumor into unaffected tissues. Thus, stable-bleb cell transformation identifies a yet unrecognized mechanism for rapid and long-range cell dispersal in vertebrates.

## Experimental Procedures

### Zebrafish Maintenance

Zebrafish (*Danio rerio*) were maintained as previously described (Westerfield, 2007). Embryos were kept in E3 medium at 25°C–31°C prior to experiments and staged based on morphological criteria (Kimmel et al., 1995) and hours postfertilization (hpf). Wild-type embryos were obtained from the Tup Longfin (TL) background unless indicated otherwise. See Extended Experimental Procedures for transgenic fish lines and *m*RNA/*morpholino* injections.

### Cell Culture

To culture primary germ layer progenitor cells, embryos were manually dechorionated in E3 buffer at sphere stage (4 hpf) or 75% epiboly stage (8 hpf). Five to ten embryos were transferred to DMEM-F12 culture medium (Sigma) and mechanically dissociated by manual tapping followed by centrifugation at 200 × *g* for 2 min. To induce stable-bleb polarization, 100 μM LPA was added during centrifugation unless indicated otherwise. See Extended Experimental Procedures for more details.

### Reagents and Inhibitor Treatments

Fetal BSA (GIBCO) and 1-Oleoyl lysophosphatidic acid (LPA, Tocris Bioscience) were used at the indicated concentrations. Fibroblast growth factor mFGF-8b and rhFGF-3 (R&D Bioscience), platelet-derived growth factor PDGF-AA (PeproTech), and epidermal growth factor hEGF (Sigma) were used at a final concentration of 100 ng/μl. Pharmacological inhibitors were used at the following concentrations: 10 μM active (−) or inactive Blebbistatin (+) (Tocris Bioscience), 100 nM Latrunculin-A (Sigma), 100 μM Y-27632 (Tocris Bioscience), 500 nM Jasplakinolide (Tocris Bioscience), 20 μM ML-141 (Sigma), and 30 μM L-294002 (Sigma). The percentage of polarized cells was measured after 15 min (Blebbistatin, LatrunculinA, and Jasplakinolide) and 30–60 min (Y-27632, ML-141, Y-294002) of exposure.

### Data Statistics

Data are presented as mean ± SEM unless indicated otherwise. Statistical tests were performed by using the Wilcoxon rank sum test in MATLAB.

## Author Contributions

V.R. and S.W. designed research, performed experiments, and analyzed the data. A.C.-J. and R.V. developed the theoretical model. M.S., H.M., and K.S. helped with in vivo experiments. V.B. generated the mezzo-GFP fishline and performed micropipette experiments. M.R.-M. and M.S. supported the work of V.R. and S.W. V.R. and C.-P.H. supervised the project and wrote the manuscript.

## Supplemental Information

Document S1. Extended Experimental Procedures

Movie S1. Motile Behavior of Embryonic Germ Layer Progenitor Cells In Vitro, Related to Figure 1 Time-lapse movie of (I) isolated progenitor cells obtained from wild-type embryos and cultured in confinement on fibronectin-coated glass substrates at serum-free conditions, (II) isolated progenitor cells obtained from *cyclops m*RNA-injected embryos to induce mesendodermal cell fate and cultured without confinement on fibronectin-coated glass substrates at serum-free conditions, and (III) isolated progenitor cells obtained from wild-type embryos and cultured in confinement on fibronectin-coated glass substrates in the presence of 20% serum. Cells were obtained from embryos at sphere stage (4 hpf).

Movie S2. Progenitor Cell Polarization upon LPA Stimulation, Related to Figures 2 and S2 Time-lapse movie showing (I) polarization of progenitor cells obtained from wild-type embryos at sphere stage (4 hpf) and cultured in suspension in the presence of 100 μM LPA. Imaging was performed 1 min after cells were added to LPA-containing culture medium. (II) Bright-field and fluorescence time-lapse movie of Lifeact-GFP (actin) localization in a polarized stable-bleb cell (4 hpf) upon application of 500 nM Jasplakinolide to the culture medium in the presence of 30 μM LPA.

Movie S3. Stimulation of Cortical Contractility via LPA Triggers a Reversible Mesenchymal-to-Amoeboid Transition, Related to Figure 2 Bright-field and fluorescence time-lapse movie of Myl12.1-eGFP (myosin II) localization during polarization of mesenchymal mesodermal progenitor cells cultured on 2D adhesive substrates and stimulated with 100 μM LPA. The mesenchymal-to-amoeboid transition (MAT) into the stable-bleb phenotype is reversible upon application of 50 μM Blebbistatin.

Movie S4. Cortex Remodeling during Progenitor Cell Polarization upon LPA Stimulation, Related to Figure 3 Time-lapse movie of Myl12.1-eGFP (myosin II) localization during polarization of progenitor cells obtained at sphere stage (4 hpf) and cultured in suspension on passivated PEG-coated glass substrates in the presence of 50 μM LPA.

Movie S5. Actin and Myosin II Localization in Migrating Stable-Bleb Cells In Vitro, Related to Figure 6 Time-lapse TIRF imaging movie showing dynamic cortex organization in motile stable-bleb cells expressing Myl12.1-eGFP (myosin II) and Lifeact-GFP (actin) obtained from embryos at sphere stage (4 hpf) and cultured in confinement on adhesive fibronectin-coated glass substrates in the presence of 30 μM LPA.

Movie S6. Migration of caRhoA-Expressing Stable-Bleb Cells In Vivo, Related to Figure 7 (I-II) Two-photon time-lapse movies of transformed stable-bleb cells migrating in the 3D tissue environment of *Tg*(*actβ1:myl12.1eGFP*) transgenic embryos with mosaic expression of caRhoA plus GPI-RFP (membrane, red) to mark caRhoA expressing cells. Scale bars represent (I) 20 μm and (II) 10 μm.

Movie S7. Fast and Persistent 3D Amoeboid Cell Motility in an Unperturbed Wild-Type Embryo, Related to Figure 7 Bright-field time-lapse movie of a wild-type AB embryo during gastrulation (∼70% epiboly stage); dorsal view. Fast migrating amoeboid cells are highlighted by colored tracks (mean instantaneous cell speed of indicated tracks <v> = 9.08 μm/min).

Movie S8. Progenitor Cell Transformation into Stable-Bleb Cells upon Wounding In Vivo, Related to Figure 7 Two-photon time-lapse movies of motile stable-cells emerging from tissue transplants using (I) a wild-type host and a *Tg*(*actβ1:myl12.1eGFP*) donor embryo expressing myosin II (green) plus GPI-RFP *m*RNA (membrane, red) and (II) a wild-type host expressing histone-mCherry (nuclei, red) and a *Tg*(*actβ1:myl12.1eGFP*) donor embryo expressing myosin II (green) plus GPI-RFP (membrane, red). Tissue transplantations were performed at sphere stage (4 hpf). Scale bars represent 50 μm. (III) Two-photon time-lapse movie of retrograde Myl12.1-eGFP (myosin II) flow in a stable-bleb cell transplanted from a *Tg*(*actβ1:myl12.1eGFP*) donor embryo into a wild-type host.

Document S2. Article plus Supplemental Information
