# Supplementary material for: Cortical Contractility Triggers a Stochastic Switch to Fast Amoeboid Cell Motility
Source: Cell. 2015 Feb 12;160(4):673–85. doi: 10.1016/j.cell.2015.01.008 (PMC4328143; doi:10.1016/j.cell.2015.01.008)
Supplement: Document S1. Extended Experimental Procedures [file mmc1.pdf]

Cell, Volume 160
Supplemental Information

Cortical Contractility Triggers a Stochastic

Switch to Fast Amoeboid Cell Motility

Verena Ruprecht, Stefan Wieser,[1,3,5] Andrew Callan-Jones, Michael Smutny, Hitoshi Morita, Keisuke Sako, Vanessa Barone, Monika Ritsch-Marte, Michael Sixt, Raphaël Voituriez, and Carl-Philipp Heisenberg

Extended Experimental Procedures

Transgenic fish lines

The following transgenic lines were used: *Tg(actβ1:lifeact-GFP)* and *Tg(actβ1:myl12.1-eGFP)* (Behrndt et al., 2012; Maître et al., 2012). The Tol2/Gateway technology (Kwan et al., 2007; Villefranc et al., 2007) was used to generate the *Tg(mezzo:eGFP)* transgenic line expressing eGFP under the control of the *mezzo* promoter. A region encompassing 2kb upstream of the transcription start side was amplified from zebrafish genomic DNA using sequence specific primers with additional Gateway recombination arms (5'–GGGGACTTTTTGTACAAACTTGACACATCTAAGGAAAAAAGTCA – 3' and 5' – GGGGACAACTTTGTATAGAAAAGTTGCATCACAACGGGTTATGAAT – 3'). The resulting PCR product was recombined with the pDONRP4-P1R (Chien#219) and subsequently with the pDestTol2pA2 (Chien#394), pME-EGFP (Chien#383) and p3E-polyA (Chien#302) vectors to obtain the pTol2-mezzo:eGFP vector. This vector was then injected in wild type TL embryos together with *m*RNA encoding for a transposase (Invitrogen).

*m*RNA injections

*m*RNA was synthesized using the mMessage mMachine Kit (Ambion). For ubiquitous *m*RNA overexpression, 100 pg *GPI-RFP*, 100 pg *ezrin-YFP*, 50 pg *n-cadherin-GFP*, or 100 pg *paxillin-GFP* (Iioka et al., 2007) *m*RNA was injected into 1-cell stage wild type, *Tg(actβ1:lifeact-GFP)* or *Tg(actβ1:myl12.1-eGFP)* embryos. To visualize the localization of integrin we used a Bimolecular Fluorescence Complementation (BiFC) construct described previously (Jülich et al., 2009) and injected 50 pg *integrinα5-nVenus* and 50 pg *integrinβ1-cVenus* into 1-cell stage wild type embryos. *e-cadherin* expression was down-regulated by using *morpholino* antisense oligonucleotides (MO; Gene Tools) injected into 1-cell stage embryos as described previously (Montero et al., 2003). For studying mesendodermal cell migration *in vitro*, 1-cell stage wild type or *Tg(actβ1:lifeact-GFP)* embryos were injected with 100 pg *cyclops m*RNA. For *in vivo* imaging of stable-bleb cells, 10 pg of constitutively active RhoA (*caRhoA*) and 100 pg of membrane RFP (*mRFP*) *m*RNA either with or without *e-cadherin* MO were injected into a single blastomere of 8 to 16-cell stage *Tg(actβ1:myl12.1-eGFP)* transgenic embryos to achieve mosaic expression. For tissue surface transplantation experiments, 1-cell stage *Tg(actβ1:lifeact-GFP)* transgenic donor embryos were injected with 100 pg *GPI-RFP* *m*RNA. Wild type host embryos were injected with 100 pg *histoneH2A-mCherry m*RNA. All

progenitor cells expressing Myl12.1-eGFP (myosin II) and Lifeact-GFP (actin) were obtained from *Tg(actβ1:myl12.1-eGFP)* and *Tg(actβ1:lifeact-GFP)* transgenic embryos.

Cell culture and *in vitro* assays

For osmotic shock experiments, a 2 x DMEM-F12 medium (~300 mOsm/L for standard 1 x DMEM-F12 medium) was prepared and diluted with H_20 to obtain hypo- and hyperosmotic medium conditions as indicated. *In vitro* mesendoderm migration assays were established by using glass-bottom dishes (MatTek) that were coated with fibronectin by adding 50 μl of 200 μg/ml bovine fibronectin (Sigma), air drying of the solution at RT and covering with 50 mg/ml BSA (Invitrogen) for 10 min. Cells were left to adhere on fibronectin-coated glass substrates for 60-90 min prior to imaging. Under-agarose migration assays were established as described previously (Renkawitz et al., 2009). Briefly, glass bottom dishes (MatTek) were pretreated with a plasma cleaner (PDC-002 Harrick) and subsequently coated with 0.2 mg/ml of PLL-PEG (Susos) overnight at 4°C or 50 μg/ml fibronectin (Sigma) for 1 h at RT. Dishes were modified with a plastic ring to delimit the inner sample chamber. 500 μl of 1 % agarose (Sigma) in DMEM-F12 (Invitrogen) containing 30 μM LPA or 20 % serum were transferred into the inner chamber. Samples were kept at RT for 5 min and then stored at 28°C with 2 ml distilled water in the outer chamber for 10 min. Around 200 cells were injected under the agarose using a small 0.2-1.0 μl pipette (Eppendorf). 2D planar glass-glass confinements were custom built using plasma-treated glass slides coated with either Poly-L-Lysine (Sigma) for 1 h at RT, 50 mg/ml BSA, 0.2 mg/ml PLL-PEG or 50 μg/ml fibronectin as indicated above. 2 μl of cell suspension were mixed with 2 μl medium containing 15 μm latex beads (Life Technologies) for separation of the glass surfaces. The sandwiched glass cover slips were rapidly sealed with wax and cell motility was imaged for 30-45 min at 28°C. For monitoring dissociated embryonic cells in suspension, cells were plated on culture dishes (MatTek) with PEG-coated surfaces prepared as described above.

Transplantation experiments

For tissue surface transplantation experiments, donor and host embryos were dechorionated in Danieau's solution [58 mM NaCl, 0.7 mM KCl, 0.4 mM $MgSO_4$, 0.6 mM $Ca(NO_3)_2$, 5 mM HEPES (pH 7.6)] and transferred to Ca^{2+}-free Ringer's solution [116 mM NaCl, 2.9 mM KCl, 5 mM HEPES (pH 7.2)]. Their EVL cells and deep cells were excised with forceps. The donor cells were attached to the host embryo in Danieau's solution. For transplantation of caRhoA plus mRFP expressing cells, mRFP labeled cells in a transgenic *Tg(actβ1:myl12.1-eGFP)* donor embryo at sphere stage were identified under a stereo-microscope and transferred into a fine glass transplantation needle (20 μm diameter). The removed cells were subsequently transplanted into a wild type host embryo at 30 % epiboly below the EVL close to the animal pole.

Micropipette assays

LPA was locally applied using a glass micropipette connected to a Microfluidic Flow Control System (Fluigent, Fluiwell), with negative pressure ranging from 7-750 Pa, a pressure accuracy of 7 Pa and change rate of 200 Pa.s^{-1}. The micropipettes were prepared by pulling glass capillaries (World Precision Instrument, TW1003) with a micro-needle puller (Sutter Instrument, P97 Flaming Brown micropipette puller) using a customized program (Ramp +3-5, Pull 50, Velocity 50, Time 50 and Pressure 500) to produce capillaries with a 1 cm long taper. The taper of the capillary was cut to an opening of ~3-5 μm internal radius and bent to a ~ 45° angle using a micro forge (Microdata 20 instrument, MFG5). The microfluidic setup was mounted on a micromanipulator (Eppendorf, Transferman Nk2). Micropipette movement and pressure were controlled via a custom programmed Labview (National Instruments) interface. For the generation of local diffusion gradients, the micropipette was loaded with 100 μM LPA (Tocris Bioscience) in PBS or 500 μg/ml Dextran tetramethylrhodamine (Invitrogen) diluted in PBS (control). The Microfluidic Flow Control System was set to have no flow in the micropipette, to allow simple diffusion of LPA. The micropipette was quickly positioned at ~5 μm distance from a cell and the response to the LPA was monitored for the following 4 min. Imaging was performed with a Leica SP5 confocal microscope equipped with a resonant scanner, using a 20 x, 0.7 NA objective (Leica) and 63 x, 1.4 NA objective (Leica). Temperature in the dish was kept constant at 28°C by a heating sample holder. To avoid medium evaporation, the sample was covered with mineral oil. For measuring temporal myosin II accumulations at the cortex upon LPA stimulation, the pipette was loaded with 10 μM LPA in PBS, cells were flushed with the solution and response was imaged for 3 min. To monitor reversible cell polarization upon LPA stimulation, progenitor cells were flushed with a 100 μM LPA solution for 2 min before the micropipette was quickly moved to a distant sample position to allow for LPA dilution. The cellular response and myosin II localization was imaged for up to 5-10 min.

Imaging

Fluorescence imaging of cells was performed on a spinning disc system (Andor Revolution Imaging System; Yokogawa CSU-X1) build on an inverted microscope (Axio Observer Z1 Zeiss) using a 63 x, 1.2 NA water immersion lens (Zeiss). The setup was equipped with a motorized piezo stage, stage heating and objective heater units. Single and dual color fluorescence images were acquired using 488 nm and 561 nm laser lines with 30 mW maximal output power and an iXon DU-897-BV EMCCD camera (Andor Technology) with exposure times set to 100-200 ms and frame rates between 1-2 s. High-resolution imaging of cortical flows was performed by Total Internal Reflection Fluorescence Microscopy (TIRFM) on an automated TIRF/Epi-fluorescence microscope (Visitron Systems) equipped with an inverted microscope (Axio Observer Z1 Zeiss) and 488 nm and 561 nm laser lines.

Images were acquired using a 63 x oil immersion objective, 1.4 NA (Zeiss) or a 100 x oil immersion objective, 1.4 NA (Zeiss) and an EMCCD camera (Evolve, Photometrix) with frame rates between 50-500 ms and exposure times of 20-50 ms. For *in vivo* fluorescence imaging, embryos were mounted at sphere stage (4 hpf) in 0.6 % agarose and subsequently imaged on an upright multiphoton microscope (TrimScope II, LaVision) equipped with a W Plan-Apochromat 20 x, 1.0 NA dipping lens (Zeiss). GFP and RFP fluorescence were imaged at 900 nm and 1100 nm excitation wavelength using a Ti-Sapphire femtosecond laser system (Coherent Chameleon Ultra) with optical parametric oscillator (Coherent Chameleon Compact OPO) technology. Excitation intensity profiles were adapted to tissue penetration depth. Z-sectioning was set between 2-7 μm. Bright-field *in vivo* imaging was performed on an upright confocal system (Leica SP5) equipped with a 25 x, 0.95 NA water dipping lens (HCX IRAPO L) and a motorized stage for multi-position imaging. *In vivo* imaging of tissue surface transplantations was additionally performed on an inverted confocal microscope, equipped with a 40 x Apochromat, 1.2 NA dipping lens. *In vitro* time-lapse bright field movies were recorded on Leica DM IL LED microscopes equipped with a custom-built temperature control system. For long-term imaging of migrating cells movies were acquired for up to 3 h with a frame rate of 10 s. All setups were equipped with a temperature control unit set to 28°C.

Data Analysis

Myosin/Actin/GPI accumulation and relative enrichment at the cortex

Cortical actin and myosin II accumulations in progenitor cells upon exposure to various LPA concentrations were measured from fluorescence line profiles with a width of 10-20 pixel in the equatorial cross-section of cells using Fiji (Schindelin et al., 2012). Fluorescence line profile data were manually analyzed and processed by custom-written algorithms in MATLAB (MathWorks, Natick, MA) to obtain the average cytosolic fluorescence F_{cyto} and average fluorescence intensity maxima at the cortex F_{max}. F_{max} was calculated as the mean over the three highest intensity values along the fluorescence intensity profiles. Data were background corrected and normalized to $(F_{max}-F_{cyto})/F_{cyto}$.

Bleb size distributions

Bleb sizes were manually measured in the equatorial cross section of the cell using Fiji. Bleb size areas were normalized to the cross-sectional area of the cell.

Cortical density profiles

Cortical actin and myosin II density profiles in polarized stable-bleb cells were measured by using progenitor cells expressing Lifeact-GFP (actin) and Myl12.1-eGFP (myosin II) obtained from sphere stage (4 hpf) *Tg(actβ1:lifeact-GFP)* and *Tg(actβ1:myl12.1-eGFP)* transgenic embryos, respectively. The cortical network was visualized by TIRFM in the contact region of polarized progenitor cells to the substrate. PEG-coated glass substrates with confinement under agarose were used to minimize cell movement during image acquisition. To extract density profiles, 50 successive frames showing minimal bleaching over the cell contact area were averaged. Fluorescence intensity values were extracted along the cell polarization axis in a rectangular region with a mean width of 10-20 pixel using Fiji. To correct for differences in contact area sizes, we used simultaneously recorded bright field images and normalized the fluorescent intensity profile along the axis of polarization to the total front to rear cell length. Fluorescence intensities were background corrected and normalized to the maximum intensity value.

Cortical actomyosin flow profiles

1-dimensional (1D) retrograde cortical flow speeds were extracted from TIRFM data by analyzing kymographs along the axis of cell polarization. Kymograph tracks were subdivided into a segmented line representation to obtain x and y positions using Fiji and further processed by custom-written algorithms in MATLAB (MathWorks, Natick, MA) to derive flow velocities. We used simultaneously recorded bright field images for outlining cell borders. Front-to-rear cell distances were normalized to total cell length for calculation of average cortical flow velocity profiles over many cells. 2D average cortical flow maps were reconstructed using TIRFM data of polarized progenitor cells from *Tg(actβ1:myl12.1-eGFP)* transgenic embryos dissociated at sphere stage and cultured on PEG-coated substrates. Imaging was performed at a frame rate of t_{lag}=100 ms. A custom-written algorithm in MATLAB was used to automatically detect bright speckles in the image stack using a predefined intensity threshold. A 7 x 7 pixel region around fluorescence intensity maxima was selected to obtain an array of sub-images that was subsequently fitted with a 2D normal distribution yielding the center of mass position (x_0,y_0), the signal width w, the number of collected photons n and local background noise. Speckle movement was tracked over time using a custom-written algorithm in MATLAB adapted from (Wieser and Schütz, 2008). Briefly, speckle positions were compared between consecutive images i and $i+1$ and connected if speckles appeared in frame $i+1$ within a predefined threshold radius $r < R_{max} = 0.5\ \mu m$ around the speckle position in frame i. Trajectories were terminated if more than one speckle position was encountered in the subsequent frame $i+1$. Calculated speckle tracks were further analyzed for frame-to-frame speed and directionality and averaged for each voxel to generate a binned 2D cortical flow map in the front of polarized stable-bleb cells. Cells were

normalized to total cell length and rotated along the main axis to create an average cortical 2D flow map.

Average cell shape

Cell shapes of polarized cells were obtained by manually tracking cell outlines obtained from spinning disc fluorescence images at the equatorial cross section. Measured x and y coordinates were interpolated by spline fitting and normalized to cell contour length using custom-written algorithms in MATLAB. All cell shapes were aligned to the origin and rotated along the main axis before calculating an average cell shape.

Filament order parameter

The cortical actin meshwork was visualized in polarized cells obtained from *Tg(actβ1:lifeact-GFP)* embryos cultured on PEG-coated glass slides in confinement under agarose using TIRF illumination. The cell polarization axis was rotated and oriented along the x-axis. Actin filaments were segmented into short straight lines from which the angle Θ to the cell polarization axis was determined using FIJI. Each angle Θ was attributed to a spatial x coordinate along the cell main axis and normalized to cell length using a bright field image as a reference. The average filament order parameter $\widetilde{Q} = \langle \cos(2\Theta) \rangle$ as a function of normalized cell length (30 % to 90 % total cell length along cell main axis) was calculated in MATLAB.

Cell tracking

Cell tracking was performed in Fiji using the "Manual Tracking Plugin" on images with a cell density less than 1 cell per 30000 μm^2. Cells were tracked for at least 0.5 h at a time interval of t_{lag}=30 s. Tracks were analyzed for cell speed and persistence time using custom-written algorithms in MATLAB. Mean square displacements were calculated according to $MSD(t_{lag})=\langle(\delta\mathbf{r}(t_{lag}))^2\rangle$ with $\delta\mathbf{r}(t_{lag})=\mathbf{r}(t+t_{lag})-\mathbf{r}(t_{lag})$ and variance $\mathrm{var} = (4Dn\Delta T)^2/(N-n+1)$ with N the number of data at the respective frame rate t_{lag} and n=2 for the two dimensional case. Data were fitted according to Fürth's formula $MSD=4D(t_{lag}-P_t(1-\exp(-t_{lag}/P_t)))$ to obtain the persistence time P_t. Net displacements were calculated as mean displacements over time $d(tlag)=\mathrm{sqrt}(\langle(\delta\mathbf{r}(t_{lag}))^2\rangle)$ with $\delta\mathbf{r}(t_{lag})$ given as indicated above.

3D image stacks

3D images were processed and analyzed using Fiji, Imaris software (Bitplane) and custom-written software in MATLAB.

Supporting Theory

1 Introduction

We present a theoretical model that accounts for the switch observed upon increase of contractility in zebrafish cells from a non polarized state to a polarized state with cortex density gradients, flows, and cell shape change. The mechanism that we propose is based on a contractility-generated instability of the cortex, described as an active gel. We start by outlining a model of cortical instability induced by myosin activity, causing spontaneous symmetry breaking of the cell from an homogeneous to an inhomogeneous state. We then describe how, in the polarized state, tension and flows in the cortex can deform the cell from its initial spherical state. We also discuss quantitatively the role of actin filament alignment in the determination of cell shape. We finally present a simplified, one-dimensional model of retrograde cortical flow in the polarized cell, in terms of cortical viscosity and friction, allowing a distinction between slipping and migrating cells.
In presenting our model we will make use of the quantities defined in the table below:

Table S1: Definition of quantities used in model

**symbol**	**definition**
θ	polar angle measured relative to z-axis (polarization axis).
$\vec{e}_r$	unit vector pointing in the radial direction (with respect to center of sphere).
$\vec{e}_\theta$	unit vector pointing in the polar direction (i.e., tangent to lines of longitude).
$\vec{e}_\phi$	unit vector pointing in the azimuthal direction (i.e., tangent to lines of latitude).
$\vec{e}_\rho$	unit vector perpendicular to, and pointing away from, z-axis.
$\vec{e}_z$	unit vector parallel to z-axis.
ρ	density of actin filaments in cortex. For axisymmetric situations, ρ only depends on polar angle, θ.
ρ_0	actin density in uniform, static cortex.
$v(\theta)$	actin speed in cortex. For axisymmetric ρ, velocity vector is $\vec{v} = v(\theta)\,\vec{e}_\theta$.
$\tilde{Q}$	anisotropy of filament nematic order parameter tensor.
$S(\rho)$	actin turnover rate (units: kg m^{-3} s^{-1}). In general, will depend on ρ.
k_p	actin polymerization rate.
k_d	actin depolymerization rate.
$T_\parallel$	component of cortical tension acting along $\vec{e}_\theta$. Tension in the cortex is a force per unit length.
$T_\perp$	component of cortical tension acting along $\vec{e}_\phi$.
ξ	friction coefficient between actin filaments and bulk cytoplasm and/or ECM.
ζ	isotropic contractility coefficient; $\zeta > 0$.
ζ'	anisotropic contractility coefficient; $\zeta' > 0$.
Π	osmotic pressure of actin filaments in cortex. Π accounts for the compressibility of the cortex.
β	coefficient of steric interaction between filaments. Quantifies the compressibility of the cortex.
γ	coefficient of density gradient energy penalty.
η	actin viscosity. Note that this is a surface viscosity, with units Pa·s/m.
β_1, χ	constants describing dynamics of filament orientational order ($\tilde{Q}$)
λ_l	growth rate of cortical fluctuation mode indexed by integer l (mode wavelength $\sim 1/l$).
$\vec{u}$	displacement vector of a point in cytoplasm from initial position.
u	elastic strain tensor, consisting of spatial derivatives of $\vec{u}$.
σ	tensor of elastic stress acting on an element of cytoplasm. Stress is linearly related to the strain.
E	Young's modulus of cytoplasm. Elastic constant relating stress and strain.
ν	Poisson's ratio. ν ranges between -1 (perfectly compressible solid) and $1/2$ (incompressible).
T_0	membrane tension.

2 Contractility-driven cortical instability and flows

We model here the mechanism by which an initially homogeneous actin cortex with no large scale flow, as observed in a non polarized cell, becomes unstable if the myosin-generated contractile stress is sufficient. The cell is then driven to a non-homogeneous state characterized by large scale actin flows, as observed in polarized cells. The main idea underlying the instability is summarized in Figure S2A: a spontaneous fluctuation of actin density results in an imbalance in the contractile forces, $F_{\rm C}$, exerted on a small element of cortex; this causes a net flow of actin in the direction of the density gradient, which, as a result, reinforces the initial density fluctuation.

The tendency towards instability is opposed by four factors: a) actin turnover, which, by generating net polymerization in low density areas and net depolymerization in high density areas, acts to smooth out density fluctuations; b) the compressibility of the cortex, which tend to energetically oppose density variations; c) friction between the cortex and either the bulk cytoplasm or the extracellular matrix, which slows down flow; and d) cortical viscosity, which opposes flow gradients. The cortical instability will occur if the contractility is sufficient to overcome these four opposing effects.

2.1 Equations for density changes and cortical flow on a sphere

Below we consider the cortical instability of a spherical cortical layer, as in Figure S2A. Using active gel theory (Kruse et al., 2005), we write down the governing equations for the actomyosin density, ρ, and velocity, $\vec{v}$. We consider long time scales, i.e. large compared to the characteristic crosslinker unbinding time, which is of order seconds. In this regime the actin cortex can be described as a viscous fluid. Note that we make no distinction between actin and myosin densities; the experiments indicate these are proportional to one another [1].

Actin mass conservation equation:

Actomyosin mass conservation at the local level means that the rate of change of actin density in a small cortical volume is balanced by the net flux of actin through the volume and by the rate of turnover. Mathematically, actin conservation is described by the following differential equation:

$$\partial_t \rho + \nabla \cdot (\rho\, \vec{v}) = S(\rho)\,, \qquad \text{S1}$$

[1] One way to rationalize this is to write the conservation equation for the density, m_b, of actin-bound myosins: $\partial_t m_b = k_{\rm on}\,\rho - k_{\rm off}\, m_b$. If the binding rate, $k_{\rm on}$, is large, then m_b simply follows the actin density, and $m_b \simeq (k_{\rm on}/k_{\rm off})\,\rho$.

where ∂_t is the time derivative; $\nabla \cdot (\ )$ is the divergence operator; and the source term $S(\rho)$ accounts for actin turnover. The simplest model equation for $S(\rho)$ is given by

$$S(\rho) = -k_d\,\rho + k_p\,, \tag{S2}$$

which assumes a uniform reservoir of free actin monomers. In the uniform, static state, $v = 0$, $\rho = \rho_0$, and actin polymerization and depolymerization balance; therefore, $\rho_0 = k_p/k_d$.

We assume throughout that cortical flow and the actin density fluctuations are axisymmetric, and thus functions only of the polar angle, θ. For axisymmetric flows with $\vec{v} = v(\theta)\,\vec{e}_\theta$, Equation S1 simplifies to

$$\partial_t \rho + \frac{1}{R\,\sin\theta}\partial_\theta\left(\sin\theta\,\rho\,v\right) = -k_d\left(\rho - \rho_0\right)\,. \tag{S3}$$

Force balance:

Denoting the cortical tensions along the polar and azimuthal directions as $T_{||}$ and $T_\perp$, the force balance along the polar unit vector $\vec{e}_\theta$ on a small element of cortex of area $dA = R^2\,\sin\theta\,d\theta\,d\phi$ (see Figure S2A) is

$$T_{||}(\theta + d\theta)\,R\,\sin\left(\theta + d\theta\right)d\phi - T_{||}(\theta)\,R\,\sin\theta\,d\phi - T_\perp\,\cos\theta\,R\,d\theta\,d\phi - \xi\,v(\theta)\,R^2\,\sin\theta\,d\theta\,d\phi = 0\,. \tag{S4}$$

In this equation, the perpendicular tension $T_\perp$ acting on the element results in a force $T_\perp\,R\,d\theta\,d\phi$ acting towards the z-axis (akin to the compression exerted by a tightened belt around a rubber ball); the component of this force along $\vec{e}_\theta$ is thus $-T_\perp\,\cos\theta\,R\,d\theta\,d\phi$. Also, ξ is the coefficient of friction between actin and its environment (outside matrix and/or cytoplasm). In the limit that the area of the cortical patch tends to zero, the above equation turns into

$$\frac{1}{R}\,\partial_\theta T_{||} + \frac{\cot\theta}{R}\left(T_{||} - T_\perp\right) = \xi\,v\,. \tag{S5}$$

Constitutive relations:

The cortical tension has three contributions: i) active, contractile stress, that we first assume isotropic; ii) viscous terms, due to velocity gradients; and iii) a pressure term (called osmotic in polymer physics) that accounts for the finite compressibility of the gel, and originates from steric (or other) interactions between filaments. In the following, we assume that the contractile, isotropic contribution to cortical tension is proportional to ρ^2 (although another choice of power law would give similar results). Then, noting that the components of the symmetrized velocity gradient tensor are $v_{\theta\theta} = \frac{1}{R}\,\partial_\theta v$ and $v_{\phi\phi} = \frac{1}{R}\,v\,\cot\theta$, the

tension components are

$$T_{\parallel} = \frac{\eta}{R}\,\partial_\theta v + \frac{\zeta}{2}\,\rho^2 - \varPi \qquad \text{S6a}$$

$$T_{\perp} = \frac{\eta}{R}\,v\,\cot\theta + \frac{\zeta}{2}\,\rho^2 - \varPi\,, \qquad \text{S6b}$$

where η is the cortical viscosity, $\zeta > 0$ is the active (contractile) coefficient, and $\varPi$ is the (osmotic) pressure in the cortex. The pressure contains two parts: a term originating from steric or (other) interactions between filaments, which becomes important at large densities and accounts for the finite compressibility of the gel, and a term coming from the density gradient energy penalty, $\gamma(\nabla\rho)^2/2$, with $\gamma > 0$. Assuming the dominant contribution to the first part of $\varPi$ varies as ρ^3 (to ensure cortical stability for large ρ), the pressure is thus

$$\varPi(\rho) = \frac{\beta}{3}\,\rho^3 - \gamma\,\frac{\rho}{R^2\,\sin\theta}\partial_\theta\left(\sin\theta\,\partial_\theta\rho\right)\,, \qquad \text{S7}$$

where $\beta > 0$.

2.2 Linear stability analysis

The cortical instability resulting from contractility can be understood theoretically by considering fluctuations in cortical actin density and velocity relative to the homogeneous, static initial state, which models a non polarized cell. If such fluctuations tend to be damped over time, the homogeneous state is stable, and in practice a cell is expected to be non polarized. In contrast, if fluctuations grow over time, the cortex is unstable and the cell is expected to switch to a polarized state. To do this, the nonlinear differential equations, Equations S3, S5, and S6 are first linearized about this initial state, characterized by $\rho = \rho_0$, $v = 0$.

Then, combining Eqs. S5-S7, we obtain the linearized force balance equation

$$\frac{\gamma}{R^3}\partial_\theta\left(\frac{\rho}{\sin\theta}\,\partial_\theta\left(\sin\theta\,\partial_\theta\rho\right)\right) + \frac{(\zeta\,\rho - \beta\rho^2)}{R}\,\partial_\theta\rho + \frac{\eta}{R^2}\,\left(\partial_\theta^2 v + \cot\theta\,\partial_\theta v - \csc^2\theta\,v\right)$$
$$= \left(\xi - \frac{\eta}{R^2}\right)\,v\,, \qquad \text{S8}$$

which relates ρ and v.

As is typical in linear stability analysis, we next assume an exponential time dependence, $e^{\lambda t}$, for $\delta\rho = \rho - \rho_0$ and v. From Equation S3 we then obtain

$$\delta\rho = -\frac{\rho_0}{R\,(\lambda + k_d)}\frac{1}{\sin\theta}\partial_\theta\left(\sin\theta\,v\right)\,. \qquad \text{S9}$$

Anticipating that $\delta\rho \sim P_l(\cos\theta)$, where $P_l(\cos\theta)$ is the Legendre polynomial [2] with $l = 1, 2, 3, \ldots$, then inserting Equation S9 into the force balance equation, Equation S8, yields

$$\left[\frac{\eta}{R^2} - \frac{\rho_0}{R\,(\lambda + k_d)}\left(\frac{\zeta - \beta\,\rho_0^3}{R} - \frac{\gamma\,\rho_0\,l(l+1)}{R^3}\right)\right]\left(\partial_\theta^2 v + \cot\theta\,\partial_\theta v - \frac{v}{\sin^2\theta}\right)$$
$$= \left(\xi - \frac{\eta}{R^2}\right) v\,. \qquad \text{S10}$$

The above differential equation is recognized as the associated Legendre equation. It follows that the velocity eigenfunctions are

$$v(\theta, t; l) = v_l(t)\, P_l^1(\cos\theta)\ , \qquad \text{S11}$$

where the time dependence of the amplitudes $v_l(t)$ is $e^{\lambda_l t}$ and P_l^m are associated Legendre polynomials [3]. Noting the recurrence relation $\frac{1}{\sin\theta}\partial_\theta\left(\sin\theta\, P_l^1\right) = -l\,(l+1)\,P_l$, and comparing with Equation S9, we confirm that the eigenfunctions of ρ are

$$\delta\rho(\theta, t; l) = \rho_l(t)\, P_l(\cos\theta)\ . \qquad \text{S12}$$

Since the eigenvalues associated with P_l^1 are $-l\,(l+1)$, from Equation S10 we obtain the dispersion relation

$$\lambda_l = -k_d + \frac{\rho_0\,\alpha_l\,l(l+1)}{\xi\,R^2}\left[\zeta - \beta\,\rho_0^3 - \frac{\gamma\,\rho_0\,l(l+1)}{R^2}\right]\ , \qquad \text{S13}$$

where

$$\alpha_l = \left[1 + \frac{\eta}{R^2\,\xi}\left(l^2 + l - 1\right)\right]^{-1}\ . \qquad \text{S14}$$

An instability of the cortex occurs if the perturbations $\delta\rho$ and v grow with time. This arises for mode l if the growth rate λ_l is positive. A few examples of λ_l versus l are shown in Figure S2D.
The condition $\lambda_l = 0$ defines, for each l, a critical value of the contractility:

$$\zeta_c(l) = \beta\,\rho_0^3 + \frac{\gamma\,\rho_0\,l\,(l+1)}{R^2} + \frac{k_d\,\xi\,R^2\,\alpha_l}{\rho_0\,l\,(l+1)}\,. \qquad \text{S15}$$

This equation defines the stability curve as a function of mode number, shown in Figure 3B. The curve $\tilde{\zeta}_c(l) \equiv \zeta_c(l)\,R^2/(\gamma\rho_0)$ defines a neutrally stable cortex; values of the non-dimensionalized contractility $\tilde{\zeta}$ above the curve represent unstable states. To obtain this curve we have chosen, for simplicity of presentation, the following dimensionless parameter values: $\tilde{\beta} \equiv \beta\,\rho_0^2 R^2/\gamma = 0$, $\tilde{k}_d \equiv k_d\,\xi R^4/(\gamma\rho_0^2) =$

[2] The first few P_l's are $P_1(\cos\theta) = \cos\theta$ and $P_2(\cos\theta) = \frac{1}{2}\left(3\cos^2\theta - 1\right)$. The P_l's are analogous to sin and cos, but on a sphere; the larger the index l, the shorter the "wavelength".

[3] The first few P_l^1's are $P_1^1(\cos\theta) = -\sin\theta$ and $P_2^1(\cos\theta) = -3\,\sin\theta\,\cos\theta$.

4, and $\tilde{\eta} \equiv \eta/(\xi R^2) = 0.1$. In the context of cell polarization, it is in practice sufficient to consider the first mode $l = 1$. The main conclusion of this paragraph is that for $\zeta \leq \zeta_c(1)$ (low contractility) the cell remains non-polarized, whereas it becomes polarized for $\zeta \geq \zeta_c(1)$ (high contractility). In particular this analysis elucidates the dependence of the transition on friction, viscosity and actin turnover.

2.3 Cortical flow and density profile in the stationary polarized state

The growth rates (Equation S13) describe the evolution of the perturbation in ρ, v at short times after the cortical instability is triggered. At longer times, nonlinearities in the mass conservation and force balance equations become relevant, and, as a result, these equations must be solved numerically. We describe below the solution method, and determine the variation of ρ and v with θ, illustrating the agreement with experiments. In Sec. 3, these steady state solutions are used to determine the cell shape change caused by cortical tension and flow. The fitting of experimentally measured cortical flows is described in Sec. 4. Steady-states solutions for ρ, v as a function of θ in the polarized state are obtained by solving Eqs. S3 and S5. The tension components $T_{||}$ and $T_{\perp}$ are functions of ρ, v, as given by Equation S6, and the pressure Π is given by Equation S7. To solve these differential equations, the regularity conditions on a sphere, namely

$$\partial_\theta \rho(\theta = 0) = \partial_\theta \rho(\theta = \pi) = 0$$

$$v(\theta = 0) = v(\theta = \pi) = 0$$

are enforced. The initial conditions ($t = 0$ being the onset of instability) are given by imposing an arbitrary perturbation in ρ about ρ_0 (up to and including the $l = 3$ mode), and setting $v(\theta, 0) = 0$. With these boundary and initial conditions, the differential equations are solved numerically in Mathematica (Mathematica, 2010); a typical solution is shown in Figure S3F, where the following parameters were used: $\tilde{\zeta} = 3.8$, $\tilde{\beta} = 1$, $\tilde{\eta} = 0.1$, and $\tilde{k}_d = 1$. Note that the shape image was determined using the methods described in Section 3.

3 Cell shape change generated by cortical instability

We present here a model of cell deformation due to cortical tension and flow. We show that tension anisotropy, which can originate from cytoskeletal flows and actin filaments ordering, plays a key role in the determination of the observed elongated pear-like shapes. Here we treat the cytoplasm (containing microtubules, intermediate filaments, and organelles permeated by a solvent (Moeendarbary et al., 2013)) and its membrane effectively as an elastic medium. In the initial state, the cell is assumed to

be spherical, and displacements of points from their undisturbed positions, $\vec{r}$, are denoted by the vector $\vec{u}(\vec{r})$; see Figure S3E. The stress in the cytoplasm is given by the second-rank tensor σ, whose indices in polar coordinates are r and θ (assuming axisymmetry about the z-axis). Note that our approach here is perturbative: we use the cortical tension components calculated from the cortical density gradients and flow on a sphere to then determine, at first order, the resulting deformation of the spherical reference state. This scheme is thus valid for large cell stiffness or, equivalently, small cortical flow and density gradients.

3.1 Relating cell deformation to stresses at cytoplasm boundary

Forces in the flowing cortex are transmitted to the cytoplasm at $r = R$. Mathematically, the stress continuity conditions are

$$\sigma_{rr}\Big|_{r=R} = -\frac{T_{\parallel} + T_{\perp}}{R} \qquad \text{S16a}$$

and

$$\sigma_{\theta r}\Big|_{r=R} = \xi\, v\,. \qquad \text{S16b}$$

The first relation expresses Laplace's law, while the second describes friction between the cortex and the cytoplasm.

At mechanical equilibrium, in the absence of volume forces, the internal stresses, acting on the faces of any element of cytoplasm must balance:

$$\nabla \cdot \sigma = 0\,. \qquad \text{S17}$$

Assuming linear elasticity, the stress and the displacement are related by the constitutive relation,

$$\sigma_{\alpha\beta} = \frac{E}{1+\nu}\left(u_{\alpha\beta} + \frac{\nu}{1-2\,\nu}\,u_{\gamma\gamma}\,\delta_{\alpha\beta}\right)\,, \qquad \text{S18}$$

expressed here in Cartesian coordinates. In this equation, E is the Young's modulus and ν is Poisson's ratio, and we assume small strains, given by $u_{\alpha\beta} = \frac{1}{2}\left(\partial_\alpha u_\beta + \partial_\beta u_\alpha\right)$. Combining the above two equations, the displacement satisfies

$$(1-2\nu)\;\Delta\vec{u} + \nabla\left(\nabla\cdot\vec{u}\right) = 0\,, \qquad \text{S19}$$

where Δ is the Laplacian operator. Since our problem is axisymmetric, $\vec{u} = u_r\vec{e}_r + u_\theta\vec{e}_\theta$.

3.2 Numerical resolution scheme

The process to calculate a shape is as follows. First, for a particular choice of parameters, the cortical tensions and friction are calculated numerically (from either measured or calculated flow and density profile), as described in Sec. 2.3 (see Equations S6). Second, the components of the stress in the cytoplasm at the surface, $r = R$, are determined from the cortical tensions and frictions via Equations S16. These stress components are then expressed in terms of components the displacement vector, $\vec{u}$, at the surface via the elastic constitutive relations in polar coordinates,

$$\sigma_{rr} = \frac{E}{1+\nu}\left(\frac{\partial u_r}{\partial r} + \frac{\nu}{1-2\nu}\nabla\cdot\vec{u}\right) - p_0 \qquad \text{S20}$$

$$\sigma_{\theta r} = \frac{E}{2\,(1+\nu)}\left(\frac{\partial u_\theta}{\partial r} + \frac{1}{r}\frac{\partial u_r}{\partial \theta} - \frac{u_\theta}{r}\right). \qquad \text{S21}$$

In the above, p_0 is a constant Lagrange multiplier needed to enforce assumed global constraint on cell volume (Tinevez et al., 2009). These provide the boundary conditions needed to solve the differential Equation S19 for the displacement vector. This problem is then solved numerically using Mathematica. Knowing $\vec{u}$ at $r = R$ we can determine the deformed shape, since a point on the surface of the undeformed sphere at angle θ gets mapped to a position

$$\vec{r}(\theta) = \left(R + u_r\big|_{r=R}\right)\vec{e}_r + u_\theta\big|_{r=R}\,\vec{e}_\theta\,. \qquad \text{S22}$$

As an example of this approach, in Figure S3F the shape of the deformed cell is shown for a given cortical flow and density profile, calculated using the methods described in Sec. 2.3.
More quantitatively, cell shapes can be characterized by the cell aspect ratio, A, given by

$$A = \frac{2R + u_z(0) + u_z(\pi)}{2(R + \max(u_\rho(\theta))}, \qquad \text{S23}$$

where $u_z = \vec{u}\big|_{r=R}\cdot\vec{e}_z$ and $u_\rho = \vec{u}\big|_{r=R}\cdot\vec{e}_\rho$. Through A we can quantitatively understand the dependence of cell shape in the polarized state on both the cortical tension anisotropy (see Figure 4H, where the following parameter values were used: $\tilde{\zeta} = 5$, $\tilde{\beta} = 1$, $\tilde{k}_d = 1$, $\tilde{\eta} = 0.2$ (low anisotropy), $\tilde{\eta} = 0.8$ (high anisotropy), and $\nu = 1/2$) and cell stiffness, E (see Figure S3G, where the following parameter values were used: $\tilde{\zeta} = 5$, $\tilde{\beta} = 1$, $\tilde{k}_d = 1$, $\tilde{\eta} = 0.8$, $\nu = 1/2$, $\tilde{E} \equiv E\,R^3/(\gamma\rho_0^2) = 1.2$ (low stiffness), and $\tilde{E} = 12$ (high stiffness)). This analysis clearly shows that tension anisotropy (here caused by the anisotropic viscous dissipation of the actin flow) is responsible for the pear-like elongated shapes that are observed experimentally. The dependence on E is more intuitive since stiffer cells are harder to deform.

Importantly the dependence on E makes it possible to discuss the effect of changes in osmotic conditions on shape. It is well known that osmotic pressure modifies the membrane tension, T_0, according to Laplace's law. Moreover, we can show that the effect of membrane tension on the cell aspect ratio can be absorbed into a redefinition of the Young's modulus as follows:

$$E \to E + \frac{4T_0}{R}(1+\nu)\frac{(7-4\nu)}{7+5\nu}. \qquad \text{S24}$$

Since $-1 \leq \nu \leq 1/2$, the effect of membrane tension is always to increase cell stiffness. This analysis predicts that A is increased when osmolarity is increased, which qualitatively agrees with observations (see Figure S3H).

3.3 Effect of actin filament ordering on shape

Beyond actin flows, a possible mechanism yielding an anisotropic tension is the ordering of actin filaments. Following Ref. (Salbreux et al., 2009), we assume that the anisotropic active stress due to myosin activity is proportional to the anisotropic part of the (traceless) nematic order parameter tensor:

$$\tilde{Q} = Q_{\theta\theta} - Q_{\phi\phi}\,, \qquad \text{S25}$$

where the components of the tensor Q are defined as averages of the orientations of individual filaments in a small volume (De Gennes and Prost, 1993). Qualitatively, for $\tilde{Q} > 0$ filaments are on average preferentially aligned along the cell polarity axis, while they are mostly perpendicular to it for $\tilde{Q} < 0$. Then, taking the existence of filament alignment into account, the tension components are

$$T_{\|} = \frac{\eta}{R}\,\partial_\theta v + \frac{\zeta}{2}\,\rho^2 + \zeta'\,\tilde{Q} - \Pi \qquad \text{S26a}$$

$$T_{\perp} = \frac{\eta}{R}\,v\,\cot\theta + \frac{\zeta}{2}\,\rho^2 - \zeta'\,\tilde{Q} - \Pi\,, \qquad \text{S26b}$$

where $\zeta' > 0$ is a second active coefficient. Using these equations, and a dynamical equation relating $\tilde{Q}$ to flow, as described below, the steps to computing the shapes described in Sections 2.3 and 3 can be reproduced.

It is well known in the hydrodynamics of nematic liquid crystals (De Gennes and Prost, 1993), and more recently has been studied in the context of cortical flows (Salbreux et al., 2009), that a spatially varying flow of filament-like molecules can result in large-scale orientational order. We argue here that coupling of the filament order, detected in the polarized state (see experimental Figures S3I and S3J), to the actin flow favors the emergence of order perpendicular to the cell polarity axis ($\tilde{Q} < 0$). As a result, this order

leads to anisotropic tension, $T_{\perp} > T_{||}$, at the cell rear, favoring the observed pear-like shapes. We show that this mechanism of flow alignment can account for the observed profiles of $\tilde{Q}$.

In the quasi one-dimensional geometry of confined cells, with flow field $v(x, t)$, the dynamics for the filament order parameter, $\tilde{Q}(x, t)$, is given by

$$\partial_t \tilde{Q} + v\, \partial_x \tilde{Q} = -\chi\, \tilde{Q} + \beta_1\, \partial_x v\,, \tag{S27}$$

where the left hand side represents the convective time derivative of $\tilde{Q}$, χ is the inverse nematic susceptibility, and β_1 is the flow alignment parameter. At steady-state, $\partial_t \tilde{Q} = 0$, and therefore

$$v\, \partial_x \tilde{Q} = -\chi\, \tilde{Q} + \beta_1\, \partial_x v\,. \tag{S28}$$

The above equation is used to fit the experimental data for $\tilde{Q}(x)$ using an exponential fit to the measured flow profile as input. The data is given between $x = 0.3$ and $x = 0.9$ (normalized units); these values correspond, respectively, to the rear and front of the portion of the cell in contact with the PEG-coated glass substrate.

We assume that a boundary condition on $\tilde{Q}$ imposes a positive value — i.e., filaments parallel to the polarity axis — at the contact zone front. Physically, this boundary condition may arise from two sources. First, from flow-induced order, noting that it is expected that the slope of v along the flow direction is positive along the contact-free cell front (i.e., $\tilde{Q}(x = 0.9) \simeq (\beta_1/\chi)v'(x = 0.9)$). Second, in the fully polarized state, cell front-specific actin polymerization nucleators may impose a given filament direction at the front. We thus have three fit parameters, χ, β_1, and $\tilde{Q}_0 \equiv \tilde{Q}(x = 0.9)$. Finally, we obtain a good fit of the experimental data (see Figure S3K), which yields

$$\chi = -5 \pm 37\ \mu\text{m/min} \tag{S29}$$

$$\beta_1 = 0.83 \pm 0.05 \tag{S30}$$

$$\tilde{Q}_0 = 1.3 \pm 0.1\,. \tag{S31}$$

This shows that the coupling of alignment to flow is sufficient to account for the observed actin filament organization.

3.4 Cell shape calculated using cortical density data

As a check on the validity of our cell shape modelling approach, using the measured cortical actomyosin density profile as input, we calculated the cell shape, taking into account the viscous contribution to

tension anisotropy. By first calculating the cortical flow, v, that satisfies mechanical equilibrium , the cortical tension components were found, and then, second, the shape was determined. We found that the resulting cell shape is in very good agreement with observations; see Figure 4G, where the following parameter values were used: $\tilde{\zeta} = 3.8$, $\tilde{\beta} = 1$, $\tilde{k}_d = 1$, $\tilde{\eta} = 0.1$, $\nu = 1/2$, and $\tilde{E} = 2.5$.

4 Model of retrograde flow in polarized state

In this section we adapt the cortex model developed in Sec. 2 to the specific geometry of confined cells. The basic model parameters in describing the retrograde flow in the cortex are the cortical viscosity and effective friction between the cortex and its environment. By fitting the experimental flow data with the model, we can extract the relative importance of friction to viscosity, thereby allowing a distinction between slipping and migrating cells.

4.1 One-dimensional description of flowing cortex

As the cells are elongated in the polarized state, we consider a one-dimensional model for cortical flow in the polarized state. Denoting the coordinate along the cell length as x, force balance on a small element of cortex of length dx is given by

$$\frac{dT(x)}{dx} = \xi\, v(x)\,, \tag{S32}$$

where $T(x)$ is the total cortical tension and $v(x)$ is the acto-myosin velocity; see Figure S4A.
The one-dimensional constitutive relation for $T(x)$ is

$$T = \eta\,\frac{dv}{dx} + \zeta\,\rho^2 - \beta\,\rho^3\,, \tag{S33}$$

where ζ and β were defined earlier. Combining Eqs. S32 and S33 we obtain

$$\eta\,\frac{d^2v}{dx^2} - \xi\,v + \frac{d}{dx}\left(\zeta\,\rho^2 - \beta\,\rho^3\right) = 0\,. \tag{S34}$$

Comparing the first and second terms, we obtain the characteristic length scale $\ell = \sqrt{\frac{\eta}{\xi}}$; on length scales shorter than ℓ, viscosity dominates, while on larger scales, friction dominates. In addition, in the absence of the third term above, the retrograde flow speed varies exponentially: $v(x) \sim e^{\pm x/\ell}$.

4.2 Retrograde flow data fitting for slipping cells

We start by first rescaling the x coordinate in Equation S34 by the cell length, L, so that $x = L\tilde{x}$. This equation then reads

$$\frac{d^2 v}{d\tilde{x}^2} - \frac{1}{\ell_f^2}\, v = -\frac{d}{d\tilde{x}} \left(\tilde{\zeta}\, \rho^2 - \tilde{\beta}\, \rho^3 \right) , \qquad \text{S35}$$

where $\ell_{\mathrm{f}} = \sqrt{\frac{\eta}{L^2\,\xi}} = \ell/L$, $\tilde{\zeta} = \frac{\zeta\, L}{\eta}$, and $\tilde{\beta} = \frac{\beta\, L}{\eta}$. To keep the notation simple, in the following we drop the tildes from $\tilde{x}$, $\tilde{\zeta}$, and $\tilde{\beta}$.

Next, we use the experimental data for ρ to obtain the flow profile. We note that we do not attempt to predict ρ (that is, by solving a one-dimensional version of the mass conservation equation, Equation S3), since this would require more precise knowledge of the relation between actin turnover rate, $S(\rho)$, and density in the polarized state than is currently available. We thus fit the cortical actin density data (Figure S4) to a fifth-order polynomial in x, which gives us a function $\rho(x)$; $\rho(x)$ is then substituted into the right hand side of Equation S35, which can be solved numerically for $v(x)$. Since this differential equation is of second-order in x, its solution requires specification of two boundary conditions, for instance, the values of $v(x)$ at the cell front and back. From the experimental data, the values of x at the front and back are

$$x_{\text{front}} = 0.9167 \qquad \text{S36}$$

$$x_{\text{back}} = 0.0167 \qquad \text{S37}$$

and the retrograde flow speed at the back, $v_{\text{back}} = v(x_{\text{back}})$, is

$$v_{\text{back}} = 0.4133\, \mu\text{m/min}\,. \qquad \text{S38}$$

Noting that the $v(x)$ data is somewhat scattered near the cell front, we do not impose a particular value of $v_{\text{front}} = v(x_{\text{front}})$ in solving Equation S35, but rather keep v_{front} as a fitting parameter.

Thus, to summarize the fitting method, we numerically solve Equation S35 for $v(x)$, with $\rho(x)$ given by a polynomial fitting function, v_{back} given by Equation S46, and with four fitting parameters: ℓ_{f}, ζ, β, and

v_{front}. The results of the fit are given in Figure S4B and the obtained fitting parameters are given below:

$$\ell_{\text{f}} = 0.31 \pm 0.04 \qquad \text{S39}$$

$$\zeta = 100 \pm 500\ \mu\text{m/min} \qquad \text{S40}$$

$$\beta = 200 \pm 500\ \mu\text{m/min} \qquad \text{S41}$$

$$v_{\text{front}} = 140 \pm 5\ \mu\text{m/min} \qquad \text{S42}$$

The standard errors on the fit parameters ζ and β are very large, which indicates that the goodness of fit of $v(x)$ is not very sensitive to these parameters. We note, however, that $v(x)$ is implicitly dependent on contractility, since the cortical instability is necessary to begin with to generate the persistent flowing state with $v_{\text{front}} > v_{\text{back}}$.

The main result from the fit is the determination of the ratio $\ell_{\text{f}} = \ell/L$, which provides information on the relative importance of viscous to frictional dissipation in the flowing cortex. To obtain an estimate of the friction coefficient $\xi = \frac{\eta}{L^2 \ell_{\text{f}}^2}$ for PEG, we take $\eta \simeq 10^{-3}$ Pa.s.m [4], and $L \simeq 10\ \mu$m, yielding

$$\xi_{\text{PEG}} \simeq 10^8\ \text{Pa.s/m}\,. \qquad \text{S43}$$

4.3 Retrograde flow data fit for running cells

Following the same procedure as for PEG, we first fit the cortical actin density data for running cells to a fifth-order polynomial in x, which gives $\rho(x)$. With

$$x_{\text{front}} = 0.9167 \qquad \text{S44}$$

$$x_{\text{back}} = 0.05 \qquad \text{S45}$$

and

$$v_{\text{back}} = -15.2016\ \mu\text{m/min}\,, \qquad \text{S46}$$

[4]Note that η is a two-dimensional viscosity: $\eta = \eta_{3\text{D}}\, e$. Taking $\eta_{3\text{D}} \simeq 2 \times 10^3$ Pa.s for actin, and $e = 500$ nm for the cortex thickness, we get $\eta \simeq 10^{-3}$ Pa.s.m.

the fit to $v(x)$ is obtained by solving Equation S35 with four fitting parameters: ℓ_{f}, ζ, β, and v_{front}. The results of the fit are given in Figure S4C and the obtained fitting parameters are given below:

$$\ell_{\mathrm{f}} = 0.19 \pm 0.01 \qquad \text{S47}$$

$$\zeta = 1100 \pm 300 \; \mu\text{m/min} \qquad \text{S48}$$

$$\beta = 950 \pm 200 \; \mu\text{m/min} \qquad \text{S49}$$

$$v_{\text{front}} = 81 \pm 1 \; \mu\text{m/min} \qquad \text{S50}$$

Again, the standard errors on the fit parameters ζ and β are quite large, indicating that the goodness of fit of $v(x)$ is not that sensitive to these parameters.

The results for ℓ_{f} for slipping and running cells, Eqs. S39 and S47, suggest that the effective friction coefficient, ξ, for running cells is larger than for slipping ones. This is consistent with the fact that greater friction provides the necessary traction for migration. Assuming $\eta \simeq 10^{-3}$ Pa.s.m and $L \simeq 10$ μm, we obtain

$$\xi_{\text{running}} \simeq 2.8 \times 10^{8} \text{ Pa.s/m}\,. \qquad \text{S51}$$

5 Determining the actin turnover rate from density and retrograde flow data

We briefly outline here how the actin turnover rate in the cortex can be related to the cortical density and flow. For simplicity, suppose there is a cortical flow of actin directed along x, as in Figure 4A.

The F-actin current, $j(x)$, is the product of the F-actin density, $\rho(x)$, and the flow speed, $v(x)$:

$$j(x) = \rho(x)\, v(x)\,. \qquad \text{S52}$$

For steady state flow, conservation of actin mass in a cortical element of width Δx means that the difference between the current flowing out of the element at $x + \Delta x$ and the current flowing in at x is given by the turnover rate $S(x)$ times the width of the element (i.e., the amount of mass added or removed from the element is proportional to its size):

$$j(x + \Delta x) - j(x) = S(x)\, \Delta x\,. \qquad \text{S53}$$

Physically, $S(x)$ corresponds to the local rate of actin polymerization or depolymerization: $S(x) > 0$ means that there is a net polymerization at x, while $S(x) < 0$ means that there is a net depolymerization.

In the limit $\Delta x \to 0$ the above equation becomes

$$\frac{d\,j(x)}{dx} = S(x)\,, \tag{S54}$$

or, in other words,

$$\frac{d\,(\rho\,v)}{dx} = S(x)\,. \tag{S55}$$

Therefore, knowing $\rho(x)$ and $v(x)$ one can obtain the F-actin turnover rate $S(x)$.

Supplemental References

Behrndt, M., Salbreux, G., Campinho, P., Hauschild, R., Oswald, F., Roensch, J., Grill, S.W., and Heisenberg, C.-P. (2012). Forces driving epithelial spreading in zebrafish gastrulation. Science *338*, 257–260.

De Gennes, P.-G. and Prost, J. (1993). The Physics of Liquid Crystals. 2nd edition, Oxford University Press, Oxford.

Iioka, H., Iemura, S.-I., Natsume, T., and Kinoshita, N. (2007). Wnt signalling regulates paxillin ubiquitination essential for mesodermal cell motility. Nat. Cell Biol. *9*, 813–U150.

Jülich, D., Mould, A.P., Koper, E., and Holley, S.A. (2009). Control of extracellular matrix assembly along tissue boundaries via Integrin and Eph/Ephrin signaling. Development *136*, 2913–2921.

Kruse, K., Joanny, J.-F., Jülicher, F., Prost, J. and Sekimoto, K. (2005). Generic theory of active gels: a paradigm for cytoskeletal dynamics. Eur. Phys. J. E *16,* 5–16.

Kwan, K.M., Fujimoto, E., Grabher, C., Mangum, B.D., Hardy, M.E., Campbell, D.S., Parant, J.M., Yost, H.J., Kanki, J.P., and Chien, C.-B. (2007). The Tol2kit: A multisite Gateway-based construction kit for Tol2 transposon transgenesis constructs. Dev. Dyn. *236*, 3088–3099.

Maître, J.-L., Berthoumieux, H., Krens, S.F.G., Salbreux, G., Juelicher, F., Paluch, E., and Heisenberg, C.-P. (2012). Adhesion Functions in Cell Sorting by Mechanically Coupling the Cortices of Adhering Cells. Science *338*, 253–256.

Mathematica (2010). Wolfram Research, Inc., Champain, Illinois.

Moeendarbary, E., Valon, L., Fritzsche, M., Harris, A. R., Moulding, D. A., Thrasher, A. J., Stride, E., Mahadevan, L. and Charras, G. T. (2013). The cytoplasm of living cells behaves as a poroelastic material. Nat. Mater. *12,* 253–61.

Montero, J.A., Carvalho, L., Wilsch-Brauninger, M., Kilian, B., Mustafa, C., and Heisenberg, C.P. (2005). Shield formation at the onset of zebrafish gastrulation. Development *132*, 1187–1198.

Renkawitz, J., Schumann, K., Weber, M., Lämmermann, T., Pflicke, H., Piel, M., Polleux, J., Spatz, J.P., and Sixt, M. (2009). Adaptive force transmission in amoeboid cell migration. Nat. Cell Biol. *11*, 1438–1443.

Salbreux, G., Prost, J. and Joanny, J. F. (2009). Hydrodynamics of cellular cortical flows and the

formation of contractile rings. Phys. Rev. Lett. *103,* 058102.

Schindelin, J., Arganda-Carreras, I., Frise, E., Kaynig, V., Longair, M., Pietzsch, T., Preibisch, S., Rueden, C., Saalfeld, S., Schmid, B., et al. (2012). Fiji: an open-source platform for biological-image analysis. Nat. Methods *9*, 676–682.

Tinevez, J.-Y., Schulze, U., Salbreux, G., Roensch, J., Joanny, J.-F. and Paluch, E. (2009). Role of cortical tension in bleb growth. Proc. Natl. Acad Sci. USA *106,* 18581–6.

Villefranc, J.A., Amigo, J., and Lawson, N.D. (2007). Gateway compatible vectors for analysis of gene function in the zebrafish. Dev. Dyn. *236*, 3077–3087.

Wieser, S., and Schütz, G.J. (2008). Tracking single molecules in the live cell plasma membrane-Do's and Don‘t's. Methods *46*, 131–140.
