# Supplementary figures and images for: Cortical Contractility Triggers a Stochastic Switch to Fast Amoeboid Cell Motility
Source: Cell. 2015 Feb 12;160(4):673–85. doi: 10.1016/j.cell.2015.01.008 (PMC4328143; doi:10.1016/j.cell.2015.01.008)

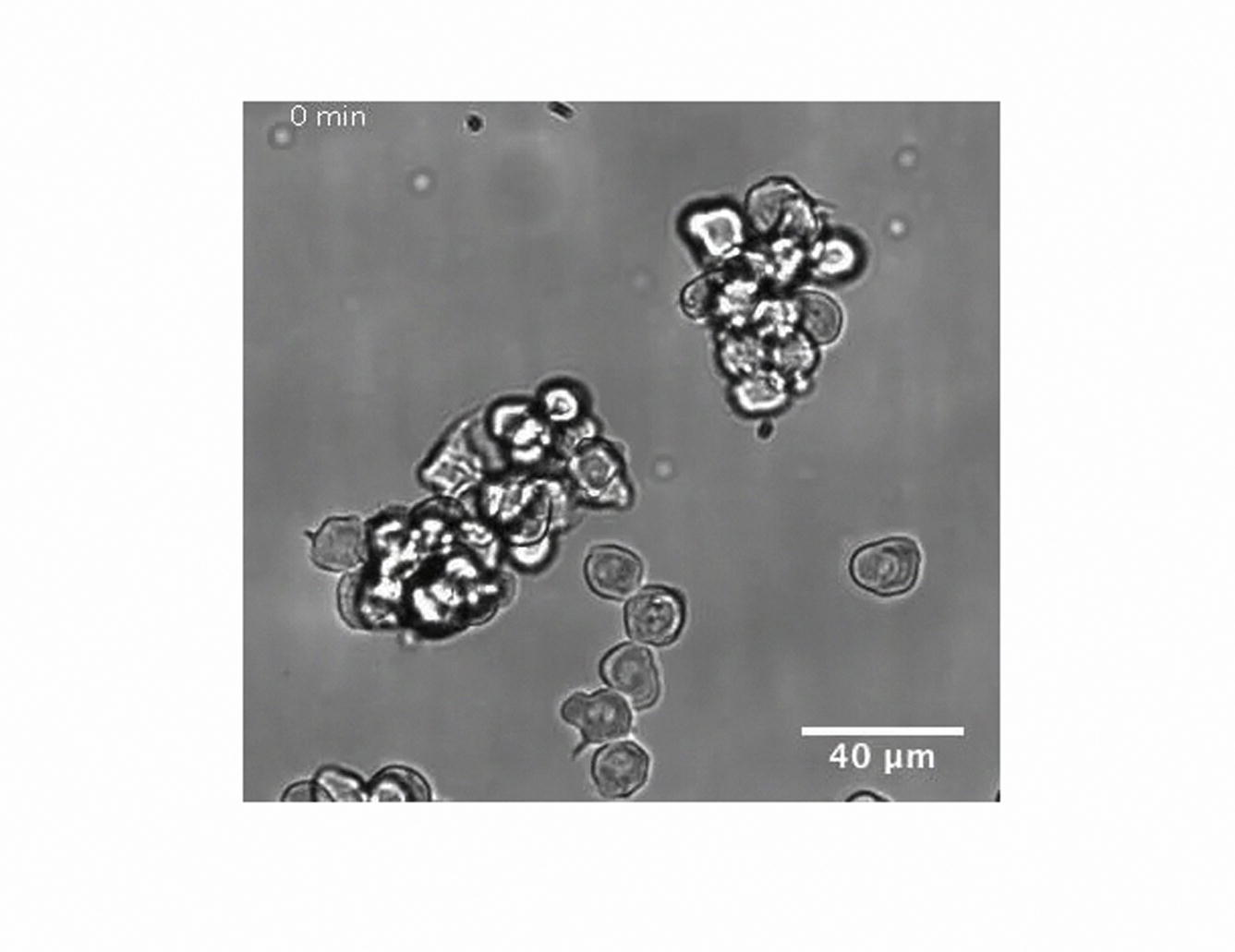

Supplement: Movie S1. Motile Behavior of Embryonic Germ Layer Progenitor Cells In Vitro, Related to Figure 1 — Time-lapse movie of (I) isolated progenitor cells obtained from wild-type embryos and cultured in confinement on fibronectin-coated glass substrates at serum-free conditions, (II) isolated progenitor cells obtained from cyclops mRNA-injected embryos to induce mesendodermal cell fate and cultured without confinement on fibronectin-coated glass substrates at serum-free conditions, and (III) isolated progenitor cells obtained from wild-type embryos and cultured in confinement on fibronectin-coated glass substrates in the presence of 20% serum. Cells were obtained from embryos at sphere stage (4 hpf). [file mmc2.jpg]

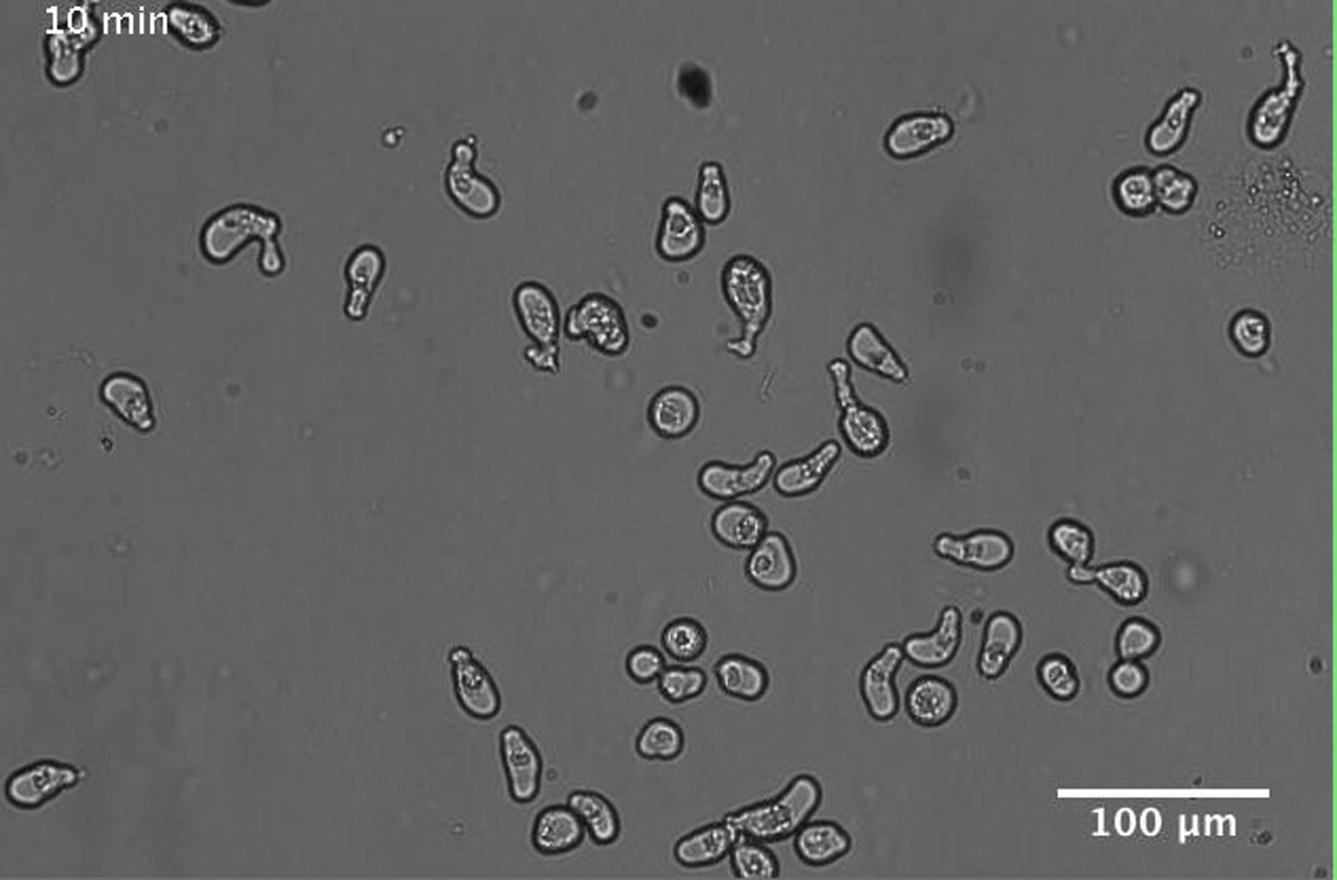

Supplement: Movie S2. Progenitor Cell Polarization upon LPA Stimulation, Related to Figures 2 and S2 — Time-lapse movie showing (I) polarization of progenitor cells obtained from wild-type embryos at sphere stage (4 hpf) and cultured in suspension in the presence of 100 μM LPA. Imaging was performed 1 min after cells were added to LPA-containing culture medium. (II) Bright-field and fluorescence time-lapse movie of Lifeact-GFP (actin) localization in a polarized stable-bleb cell (4 hpf) upon application of 500 nM Jasplakinolide to the culture medium in the presence of 30 μM LPA. [file mmc3.jpg]

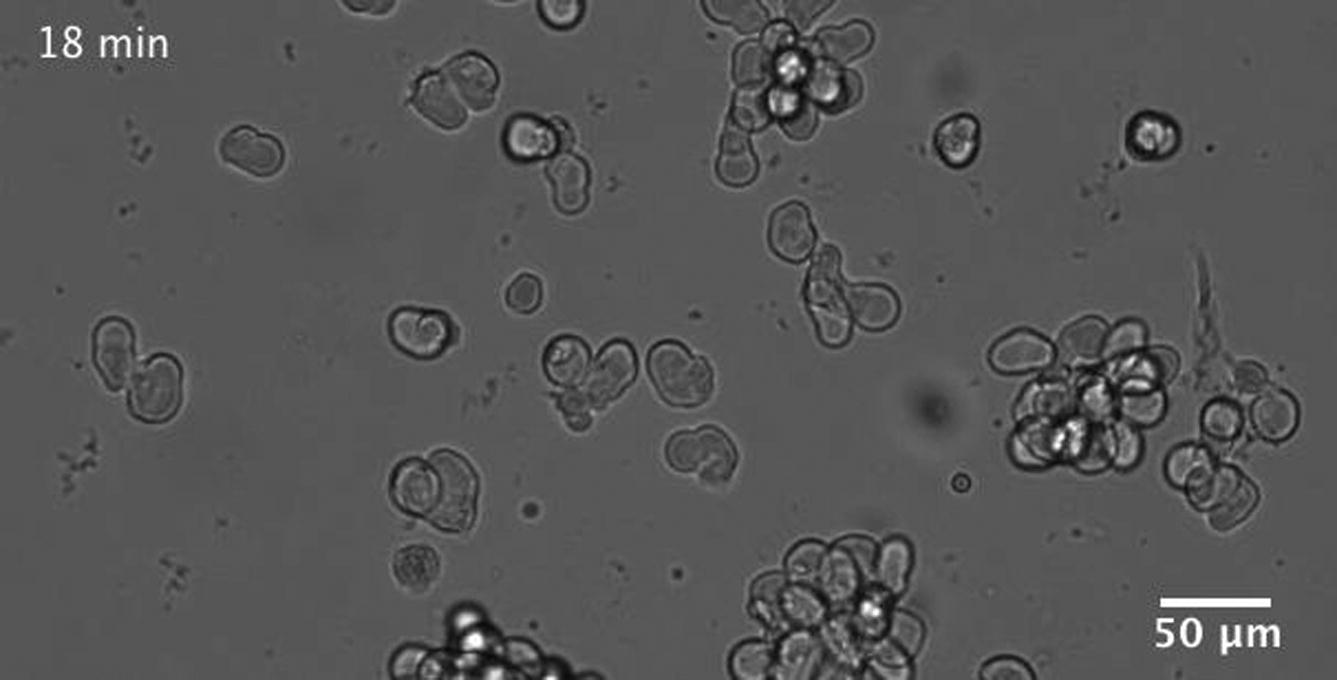

Supplement: Movie S3. Stimulation of Cortical Contractility via LPA Triggers a Reversible Mesenchymal-to-Amoeboid Transition, Related to Figure 2 — Bright-field and fluorescence time-lapse movie of Myl12.1-eGFP (myosin II) localization during polarization of mesenchymal mesodermal progenitor cells cultured on 2D adhesive substrates and stimulated with 100 μM LPA. The mesenchymal-to-amoeboid transition (MAT) into the stable-bleb phenotype is reversible upon application of 50 μM Blebbistatin. [file mmc4.jpg]

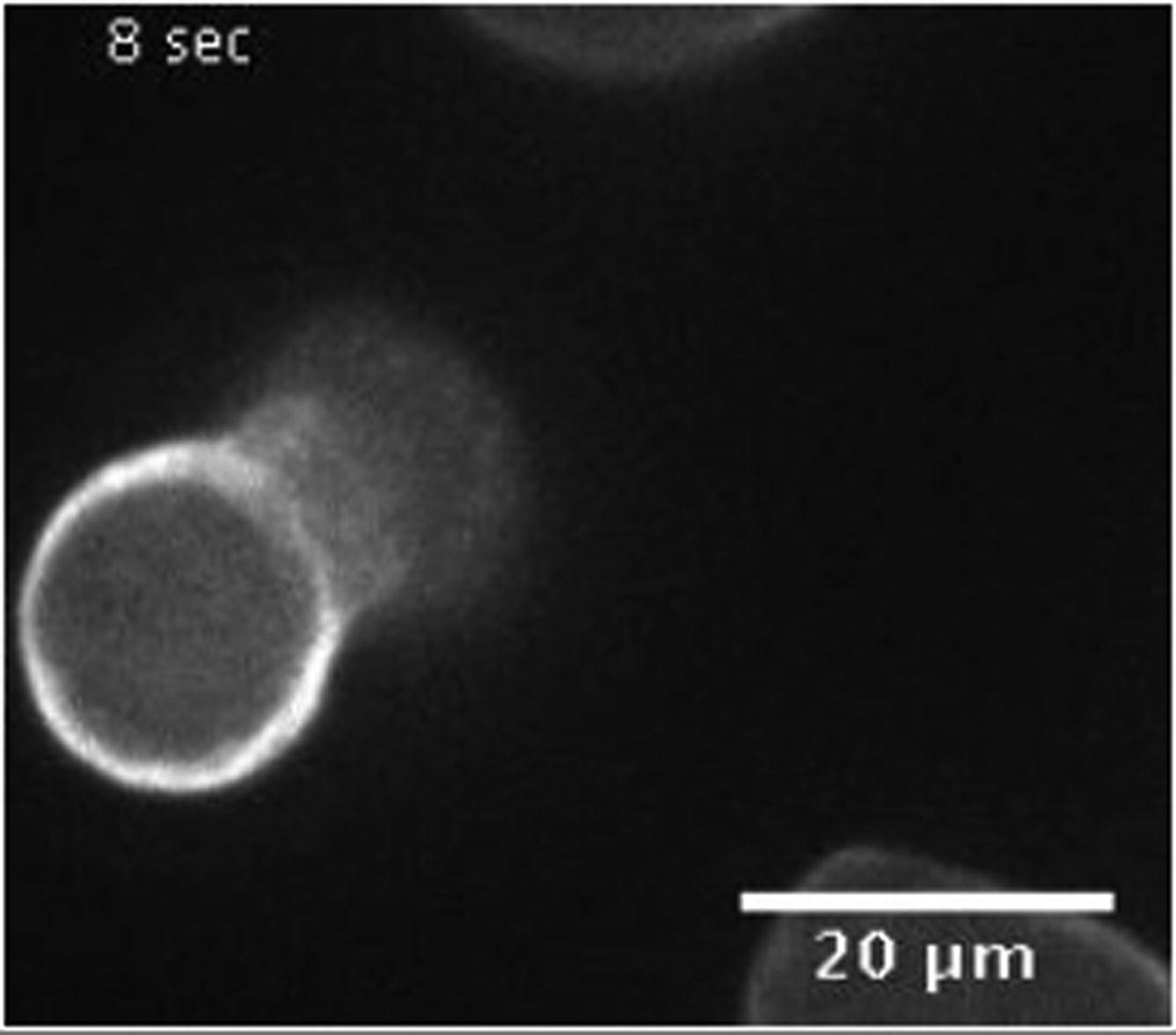

Supplement: Movie S4. Cortex Remodeling during Progenitor Cell Polarization upon LPA Stimulation, Related to Figure 3 — Time-lapse movie of Myl12.1-eGFP (myosin II) localization during polarization of progenitor cells obtained at sphere stage (4 hpf) and cultured in suspension on passivated PEG-coated glass substrates in the presence of 50 μM LPA. [file mmc5.jpg]

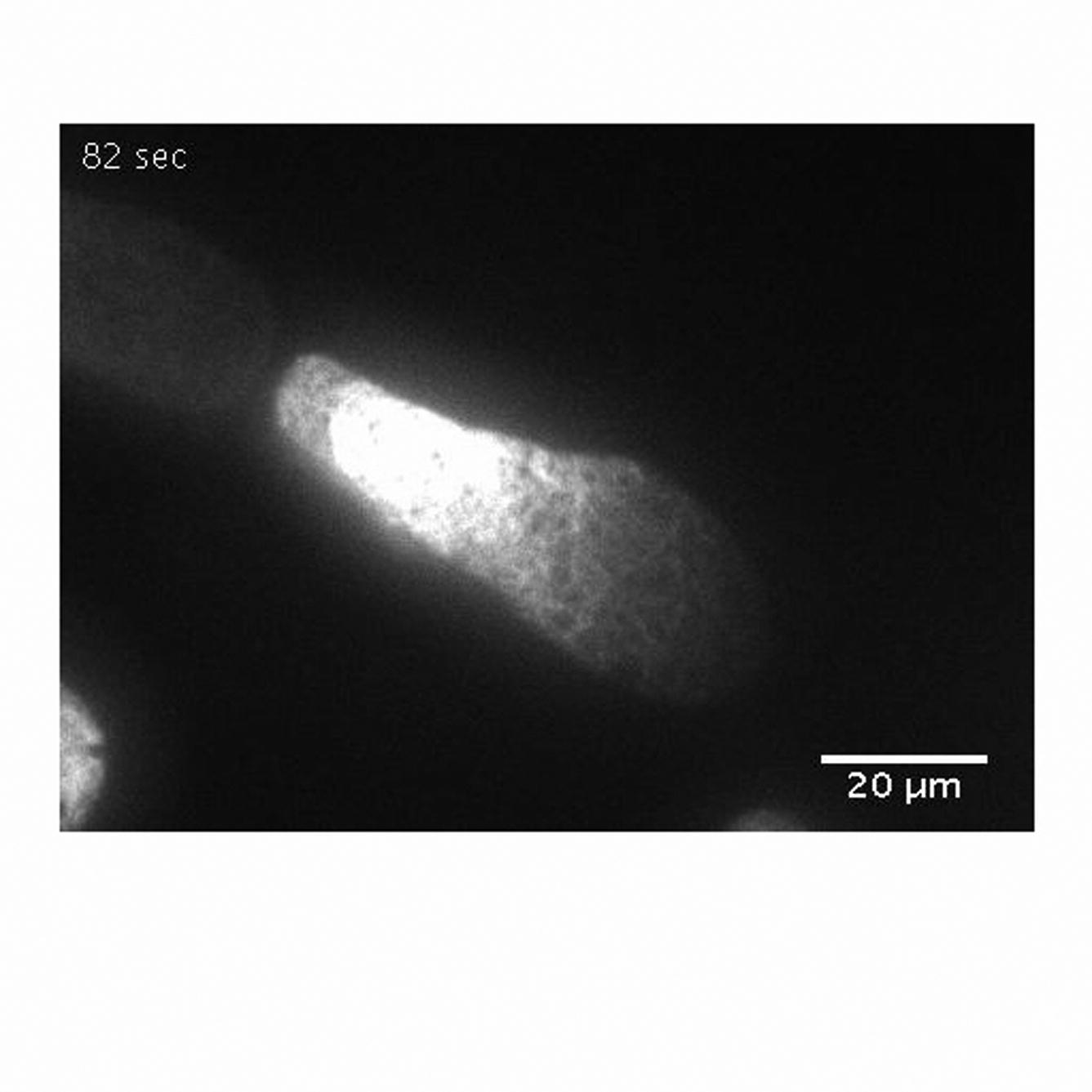

Supplement: Movie S5. Actin and Myosin II Localization in Migrating Stable-Bleb Cells In Vitro, Related to Figure 6 — Time-lapse TIRF imaging movie showing dynamic cortex organization in motile stable-bleb cells expressing Myl12.1-eGFP (myosin II) and Lifeact-GFP (actin) obtained from embryos at sphere stage (4 hpf) and cultured in confinement on adhesive fibronectin-coated glass substrates in the presence of 30 μM LPA. [file mmc6.jpg]

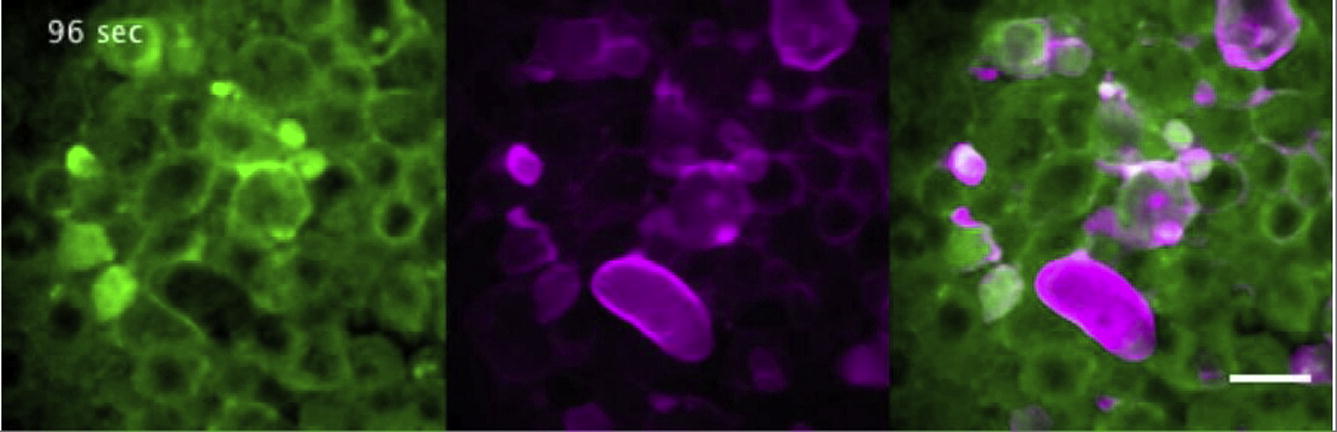

Supplement: Movie S6. Migration of caRhoA-Expressing Stable-Bleb Cells In Vivo, Related to Figure 7 — (I-II) Two-photon time-lapse movies of transformed stable-bleb cells migrating in the 3D tissue environment of Tg(actβ1:myl12.1eGFP) transgenic embryos with mosaic expression of caRhoA plus GPI-RFP (membrane, red) to mark caRhoA expressing cells. Scale bars represent (I) 20 μm and (II) 10 μm. [file mmc7.jpg]

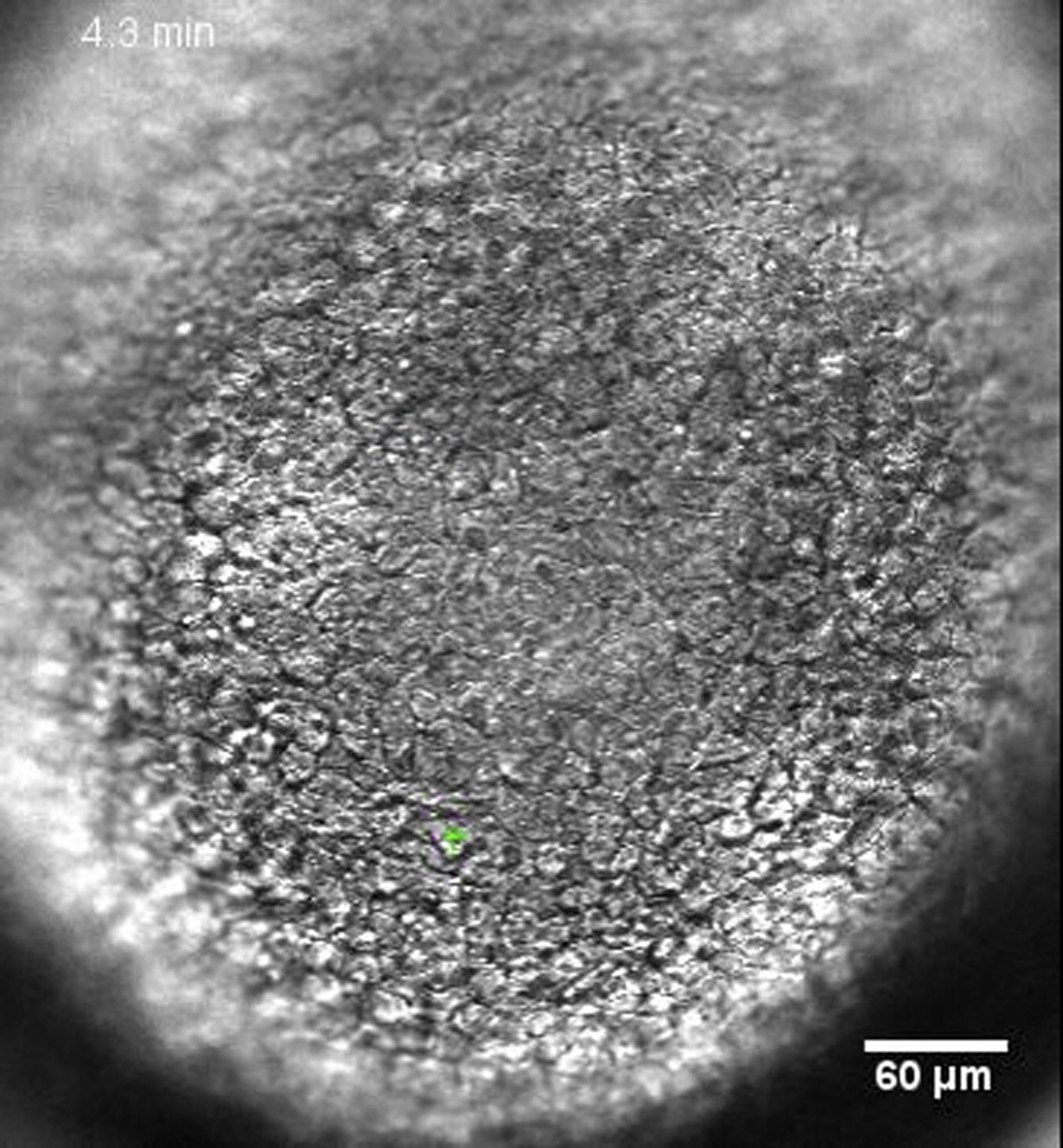

Supplement: Movie S7. Fast and Persistent 3D Amoeboid Cell Motility in an Unperturbed Wild-Type Embryo, Related to Figure 7 — Bright-field time-lapse movie of a wild-type AB embryo during gastrulation (∼70% epiboly stage); dorsal view. Fast migrating amoeboid cells are highlighted by colored tracks (mean instantaneous cell speed of indicated tracks = 9.08 μm/min). [file mmc8.jpg]

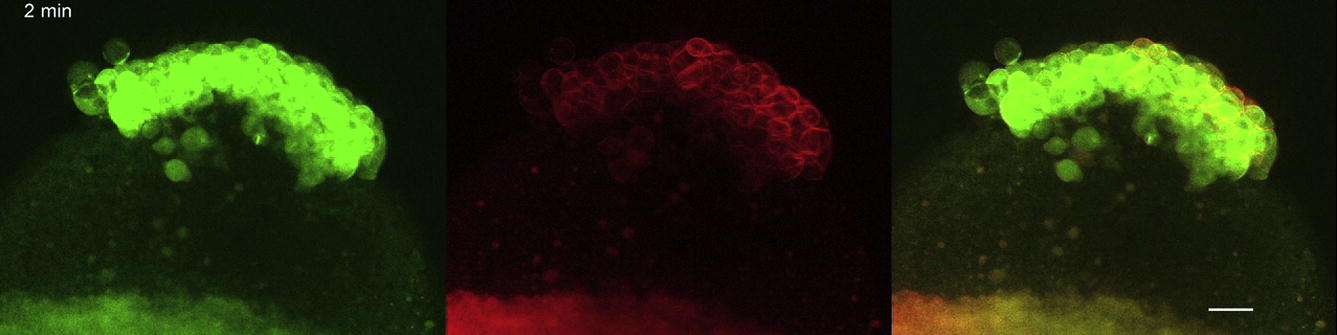

Supplement: Movie S8. Progenitor Cell Transformation into Stable-Bleb Cells upon Wounding In Vivo, Related to Figure 7 — Two-photon time-lapse movies of motile stable-cells emerging from tissue transplants using (I) a wild-type host and a Tg(actβ1:myl12.1eGFP) donor embryo expressing myosin II (green) plus GPI-RFP mRNA (membrane, red) and (II) a wild-type host expressing histone-mCherry (nuclei, red) and a Tg(actβ1:myl12.1eGFP) donor embryo expressing myosin II (green) plus GPI-RFP (membrane, red). Tissue transplantations were performed at sphere stage (4 hpf). Scale bars represent 50 μm. (III) Two-photon time-lapse movie of retrograde Myl12.1-eGFP (myosin II) flow in a stable-bleb cell transplanted from a Tg(actβ1:myl12.1eGFP) donor embryo into a wild-type host. [file mmc9.jpg]
